# Novel cardiovascular magnetic resonance strain heterogeneity phenotypes predict cardiovascular events: A prospective UK Biobank study

**DOI:** 10.1016/j.jocmr.2025.101976

**Published:** 2025-10-29

**Authors:** Kerrick Hesse, Mohammed Y. Khanji, C. Anwar A. Chahal, Sucharitha Chadalavada, Kenneth Fung, Jose D. Vargas, Jose Paiva, Steffen E. Petersen, Nay Aung

**Affiliations:** a William Harvey Research Institute, NIHR Barts Biomedical Research Centre, Queen Mary University London, Charterhouse Square, London, EC1M 6BQ, UK; b Academic Cardiovascular Unit, James Cook University Hospital, Marton Road, Middlesbrough TS4 3BW, UK; c Newham University Hospital, Barts Health NHS Trust, Glen Road, Plaistow, London E13 8SL, UK; d Barts Heart Centre, St Bartholomew’s Hospital, Barts Health NHS Trust, West Smithfield, EC1A 7BE, London, UK; e Center for Inherited Cardiovascular Diseases, WellSpan Health, York, PA; f Department of Cardiovascular Medicine, Mayo Clinic, Rochester, MN; g DC Veterans Affairs Medical Center, Washington DC

**Keywords:** Biomarker, Cardiovascular magnetic resonance imaging, Coefficient of variation, Global longitudinal strain, Heterogeneity

## Abstract

**Background:**

Global longitudinal (GLS)*,* circumferential (GCS) and radial (GRS) strains may be insufficiently sensitive to early regional pathological cardiac remodeling on cardiovascular magnetic resonance imaging (CMR). Corresponding strain coefficients of variation, CoV_LS_, CoV_CS_ and CoV_RS_, may facilitate improved prognostication by quantifying contractile heterogeneity.

**Objectives:**

To compare CoV_LS_*,* CoV_CS_, CoV_RS_, to GLS, GCS, GRS, to predict incident cardiovascular (CV) events at population level, respectively.

**Methods:**

CMR feature tracking-derived strain biomarkers from 60,746 UK Biobank participants were calculated. Kaplan-Meier survival analysis and Cox proportional hazards regression analyzed unadjusted and adjusted associations between strain biomarkers and CV outcomes, respectively. Covariables included age, sex, CV risk factors, left ventricular mass, end-diastolic volume, ejection fraction (LVEF) and pre-existent regional strain abnormalities. Logrank test for trend, hazard ratios (HR) and *Uno’s* C-Index compared relative performances of CoV and global strain.

**Results:**

Over a median follow-up 5.1 years, higher CoV_CS_, CoV_RS_ and lower GLS predicted greater risk of all-cause death (HR 1.10 [1.02–1.19]; HR 1.08 [1.01–1.16]; HR 0.84 [0.77–0.93], respectively) and a composite CV endpoint of myocardial infarction (MI), heart failure (HF) and arrhythmia (HR 1.10 [1.04–1.16]; HR 1.06 [1.01–1.12]; HR 0.77 [0.71–0.82], respectively). Model discrimination of HF and arrhythmia were significantly improved by CoV_CS_ (△C-index 0.004 [P<0.001], 0.002 [P=0.001], respectively) and CoV_RS_ (△C-index 0.004 [P<0.001], 0.001 [P=0.004], respectively). When LVEF ≥50% and ≥3 of age >65 years, obesity, hypertension, diabetes, atrial fibrillation and chronic kidney disease, CoV_CS_ and CoV_RS_ were more predictive of incident HF than GLS (HR 1.37 [1.14–1.65]; HR 1.35 [1.14–1.60]; HR 0.68 [0.53–0.86], respectively). When LVEF <50%, CoV_CS_ and CoV_RS_ were superior to GLS in predicting the composite CV endpoint, HF and arrhythmia (logrank test for trend, P<0.001 for all with CoV_CS_ and CoV_RS_ vs P=0.022, P=0.15, P=0.030 for GLS respectively).

**Conclusions:**

Heterogeneity biomarkers are sensitive to early pathological signals by measuring disease regionality. CoV_CS_ and CoV_RS_ are significant, consistent, additive and sometimes superior predictors of HF, arrhythmia and all-cause death than established risk markers*,* particularly in cohorts with multiple co-morbidities or LVEF <50%. Expansion of machine learning-guided image analysis makes strain CoV imminently translatable into routine clinical practice.

## Graphical abstract

**Figure:**
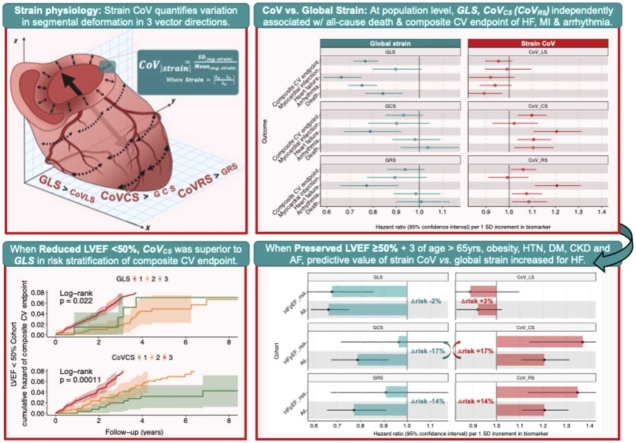
**Central Illustration. Strain coefficient of variation is a novel CMR biomarker of contractile heterogeneity.***CoV*_*CS*_ and *CoV*_*RS*_ are prognostic of future CV events. Greater *CoV*_*CS*_ and *CoV*_*RS*_ are strongly associated with HF in participants with 1) reduced LVEF < 50% and 2) preserved LVEF ≥ 50% in addition to old age, obesity, HTN, DM, CKD and AF. CoV: coefficient of variation; GLS: global longitudinal strain; CoV_LS_: longitudinal strain coefficient of variation; GCS: global circumferential strain; CoV_CS_: circumferential strain coefficient of variation; GRS: global radial strain; CoV_RS_: radial strain coefficient of variation; CV: cardiovascular; HF: heart failure; LVEF: left ventricular ejection fraction; HTN: hypertension; DM: diabetes mellitus; CKD: chronic kidney disease; AF: atrial fibrillation.

## 1 INTRODUCTION

Myocardial strain evaluation on cardiovascular magnetic resonance (CMR) and echocardiography is a noteworthy paradigm shift in the assessment of cardiac function. Compared to the established crude global measure of left ventricular ejection fraction (LVEF) and qualitative delineation of regional wall motion frequently reported on echocardiography, radionuclide studies, left ventriculography and cine CMR, myocardial strain can precisely quantify regional cardiac function. [1], [2], [3], [4], [5] Dedicated CMR sequences and post-processing algorithms measure longitudinal, circumferential and radial myocardial deformation during the cardiac cycle to derive measures of contractile function, dyssynchrony and discoordination. [1], [2], [5].

Global longitudinal (GLS) and circumferential (GCS) strains are exemplar metrics that facilitate earlier diagnosis and improved risk stratification both in the general population and across a range of cardiovascular (CV) diseases, including non-ischemic cardiomyopathies, coronary artery disease (CAD), iatrogenic cardiotoxicity and congenital heart disease.[6], [7], [8], [9], [10], [11] However, disease affects cardiac function heterogeneously, raising the concern that global strain assessment may not be sensitive and specific enough for conditions causing regional cardiac dysfunction, especially in the early stages.

**Fig. 1:**
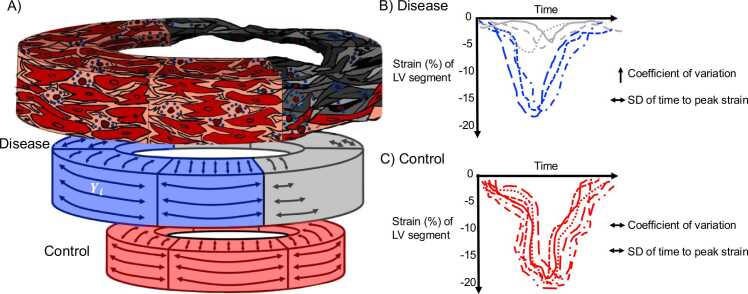
**Strain curves in ischemic heart disease versus healthy controls.** For example, anterolateral MI leads to hypokinesis of the respective LV wall, impairing regional strain and therefore increasing strain CoV without necessarily increasing the SD of time to peak strain i.e. mechanical dispersion. *SD* standard deviation, *MI* myocardial infarction.

Using speckle tracking echocardiography, multiple case-control studies have demonstrated that mechanical dispersion, defined as the standard deviation of time to peak segmental strain in a 16-segment left ventricular (LV) model is predictive of ventricular arrhythmias (VA) in cardiomyopathies and channelopathies, independent of both LVEF and global strain and may improve cardiac resynchronization therapy patient selection. [12], [13], [14], [15], [16], [17] Very importantly though, regional heterogeneity in LV systolic function is characterized by variation in not only myocardial electrical activation, but also in myocardial mechanical deformation i.e. contraction **(**Fig. 1**).** To date, the comprehensive evaluation of contractile heterogeneity assessed by segmental strain variation remains limited and underpowered. [18], [19] Reservations regarding the reproducibility of segmental strain also need further exploration. [11].

The concept of strain heterogeneity biomarkers on CMR is intuitive and promising. This study aimed to evaluate the prognostic role of novel strain heterogeneity biomarkers derived from CMR feature tracking at population level. We assessed the added independent predictive value of longitudinal (CoV_LS_*)*, circumferential (CoV_CS_*)* and radial (CoV_RS_) strain coefficients of variation in multivariable global strain-adjusted clinical risk models of CV events.

## 2 METHODS

### 2.1 Study population

In brief, the UK Biobank (UKBB) is a large prospective cohort study of over 500,000 volunteers recruited between 2006–2010 across the UK with comprehensive baseline phenotyping and ongoing outcome data accrual. [20] We accessed the UKBB resource with outcome data collected until 2024 (under application number 2964). The UKBB project was approved by the North West Multi-center Research Ethics Committee (MREC), most recently under 21/NW0157. [21] Candidate participants were identified and invited if they were aged 40–69 years, were registered with a general practitioner within the National Health Service (NHS) and lived within reasonable distance of an assessment center. [20] All participants provided written consent to participate and publish pseudonymized health-related data as part of the recruitment process. [20] The cohort's sociodemographic, anthropometric, biochemical, and clinical characteristics have been extensively summarized in previous publications, most recent available from: https://biobank.ndph.ox.ac.uk/ukb/docs.cgi?id=2&year=2025. A random subset of 100,000 participants will have CMR as part of a whole-body magnetic resonance imaging study. [22], [23] Participants with available CMR scans at the time of image analysis were included, provided valid volume-derived and strain-derived biomarkers satisfied our internal quality control as detailed below.

### 2.2 CMR imaging protocol

The UKBB CMR protocol rationale and design have been described previously. [23] In brief, after localizing sequences, balanced steady-state free precession (bSSFP) cines that included three long axis i.e. horizontal long axis, vertical long axis and left ventricular outflow tract as well as a complete short axis stack with slice thickness of 8.0 mm and slice gap of 2 mm of the left and right ventricle were acquired on a 1.5 Tesla scanner (MAGNETOM Aera, Syngo Platform VD13A, Siemens Healthineers, Erlangen, Germany). [23] Image acquisition and analysis were subject to stringent quality control by the CMR Image Analysis Consortium. [22], [23].

### 2.3 CMR post-processing and strain analysis

CMR scans were analyzed using CVI42 post-processing software (Versions 5.14.1 and 5.13.7, Circle Cardiovascular Imaging Inc., Calgary, Alberta, Canada). Standard volumetric and functional parameters were calculated with a modified automatic image analysis pipeline that was based on a fully convolutional network trained and tested against 4875 expert-annotated CMR scans with Dice metrics 0.94 for LV cavity and 0.88 for LV myocardium. [24], [25], [26], [27].

CMR feature tracking (CMR-FT) was performed to derive strain metrics. A machine learning segmentation algorithm based on a nearly incompressible LV deformation model tracked myocardial features in the region of interest, delineated by LV epicardial and endocardial contours on long and short axis cine images from end-diastole, the reference phase to end-systole with temporal smoothing. [28] Myocardial deformation was quantified in the radial and circumferential direction on short axis slices and in the longitudinal direction on long axis slices based on 2D segmented myocardium tracker position change using the Lagrangian strain tensor method. [28] Sequences with open contours and/or absent blood pool were excluded.

### 2.4 CMR biomarker quality control

The minimum dataset from each CMR scan included indexed LV mass (LVMi), end-diastolic volume (LVEDVi), end-systolic volume (LVESVi) and EF; global longitudinal (GLS), circumferential (GCS) and radial (GRS) strains; and CoV_LS_, CoV_CS_ and CoV_RS_, Per consensus reporting recommendation, strain markers were reported in absolute value. [29] Therefore, lower *GLS, GCS* and *GRS* indicated worsening global function whereas greater CoV_LS_*,* CoV_CS_ and CoV_RS_ indicated greater functional heterogeneity. GLS, GCS and GRS strains were the average of peak absolute systolic strain values from strain curves across the entire myocardium on imaging planes parallel to the respective vector direction. Corresponding coefficients of variation, CoV_LS_, CoV_CS_ and CoV_RS_, were calculated as the standard deviation (SD) divided by the mean of peak segmental strain based on the American Heart Association (AHA) 16-segment LV model without an apical cap **(**Fig. 1**).** [30] As an illustration, CoV_CS_ was calculated using the following equation:

$${\mathrm{CoV}}_{\mathrm{CS}}=\frac{{\mathrm{SD}}_{\left||,\mathrm{CS},|\right|}}{{\mathrm{Mean}}_{\left||,\mathrm{CS},|\right|}}$$

Where 1) $Y_{i}=$ circumferential strain of segment i of 16 segment LV model **(**Fig. 1**A).**

$${\mathrm{Mean}}_{\left||,\mathrm{CS},|\right|}=\frac{\left|Y_{1}\right|+\left|Y_{2}\right|+\left|Y_{3}\right|\ldots +\left|Y_{i}\right|}{16}$$

$${\mathrm{SD}}_{\left||,\mathrm{CS},|\right|}=\sqrt{\frac{\sum {\left(\left|Y_{i}\right|-{\mathrm{Mean}}_{\mathrm{CS}}\right)}^{2}}{16}}$$

To mitigate bias introduced by erroneous and incomplete data sampling, our strain quality control pipeline excluded the following:

1) Participants with no strain measurements.
2) Raw strain output that was non-sensical (e.g. positive longitudinal, circumferential, or negative radial strain) and/or ≥3x interquartile range (IQR) below 25th percentile i.e. above 75th percentile. This is in line with previous censoring of the same automated image analysis that demonstrated >95% agreement between automated and trained physician CMR contouring and segmentation. [31]
3) CoV derived from ≤6 segments, based on graphical and statistical impact assessment of missing segmental strain on CoV **(**Supplemental Fig. 1**)**.

### 2.5 Phenotype definitions

Clinical phenotype definitions were based on a combination of self-reported diagnoses, disease-specific medications, biochemical profiles as well as medical codes according to the International Classification of Diseases (ICD-9 and −10) and the Operating Procedure Codes Supplement (OPCS-3 and −4). Medical codes and date of first diagnosis were provided by the NHS in England, Scotland and Wales, respectively. [32] Supplemental Table 1 lists the definitions of cardiometabolic risk factors and cardiovascular diseases (CVD) of interest. Body mass index (BMI) ≥ 30.0 kg/m^2^ defined obesity. A predefined healthy reference cohort selected participants without prevalent cardiometabolic risk factors and CVD. Amongst others, these included dyslipidemia, obesity, hypertension, diabetes mellitus, chronic kidney disease (CKD), active smoking, CAD and myocardial infarction (MI), cerebrovascular and peripheral vascular disease (PVD), atrial fibrillation and flutter (AF), aortic stenosis (AS), mitral valve disease, cardiomyopathy and heart failure (HF).

Important outcomes were death from any cause and a composite CV endpoint that included MI, hospitalization for HF and arrhythmia. The arrhythmia endpoint included AF and VA. Hospitalization for HF included hospital admissions, coded with a diagnosis of dilated cardiomyopathy, but not hypertrophic or arrhythmogenic cardiomyopathy. Prevalent i.e. pre-existing disease was diagnosed before, and incident i.e. new disease was diagnosed after the date of CMR. Although participants could have multiple event episodes, we only considered dates of first diagnosis. [32] This approach was justified by our focus on prognosis of incident disease rather than assessment of disease progression.

### 2.6 Statistical analysis

Data were analyzed on the open-source coding software R version 4.4.0 (Vienna, Austria: R Core Team 2023, available at https://www.r-project.org). Important statistical packages included *tidyverse, lubridate, survminer, corrplot, ggforestplot, rms, rmsMD, lmtest* and *boot.*

The normality of data distribution was visually assessed by density and quantile-quantile plots **(**Supplemental Fig. 2**).** Numerical variables were reported as mean ± SD or median (Q1, Q3); categorical variables were presented as proportions. Upper and lower limits of strain reference ranges were defined as 2 SD above and below the mean values in the healthy reference cohort. The non-parametric distribution of strain CoV meant we used the Mann-Whitney *U* test to evaluate strain biomarker differences across groups, dichotomized by mean age, sex, ethnicity, CV risk factors and disease, respectively. Spearman rank correlation coefficient (ρ) quantified the correlation between CMR biomarkers, including strain CoV and global strain.

Amongst participants without prevalent disease, Cox proportional hazards regression methodology modeled the incidence of previously defined clinical outcomes against CoV and global strain biomarkers. Models were constructed and compared in a stepwise fashion, starting with **Model 1)** univariable analysis before sequentially adding **Model 2)** modifiable and non-modifiable cardiometabolic risk factors (i.e. age, sex, ethnicity, smoking status, BMI, dyslipidemia, hypertension and diabetes) and **Model 3)** other CMR biomarkers, including LVMi, LVEDVi, LVEF and the corresponding global i.e. CoV strain marker. Strain parameters were standardized to the z-distribution defined by N(0,1). Principal comparisons were CoV_LS_ to GLS*,* CoV_CS_ to GCS*,* and CoV_RS_ to GRS*.* Examination of Schoenfeld residual plots (Supplemental Figs. 3–7) and variance inflation factors (VIP) (Supplemental Table 2) demonstrated agreement with the proportional hazards assumption and absence of multicollinearity (all VIP < 5.0), respectively.

Hazard ratios (HR) and 95% confidence intervals (CI) estimated biomarker effect sizes and predictive values. *Uno’s* Concordance Index (C-Index) measured model i.e. biomarker probability to correctly discriminate randomly selected pairs of comparable individuals, one of whom either outlived (censored) or developed the outcome after the other. [33], [34] Larger values after the addition of candidate strain biomarkers suggested incremental improvement in overall model discriminative performances. Differences in C-index before and after biomarker inclusion were evaluated by an unpaired *t* test, using the bootstrap resampling methodology. The continuous net reclassification improvement (NRI(>0)) quantified the model’s percent correctness in re-ranking participants in lower and higher risk directions. [35], [36] The Brier score assessed model calibration by comparing predicted risk to observed outcomes at 5 years with a lower Brier score indicative of a more accurate model. Finally, the Likelihood ratio test (LRT) and Akaike information criterion (AIC) evaluated overall model performance. [36] Lower AIC and statistically significant improvement in the chi-square (χ^2^) estimate by LRT after the addition of biomarkers indicated an overall more robust model, respectively.

We performed the following sensitivity analyses, using **Model 3**:

1) an analysis in predefined subgroups of participants, stratified by age and sex as well as with prevalent CKD, MI, AF and at increased risk of heart failure with preserved ejection fraction (HFpEF), respectively. Participants with preserved LVEF ≥ 50% and at least 3 criteria of age > 65 years, obesity, hypertension, diabetes, CKD and AF were considered at increased risk of HFpEF; [37]
2) an analysis with restricted-cubic-spline-transformed (RCS) strain CoV biomarkers to investigate non-linear relationships with outcomes. [38] Our RCS transformations had 4 knots, located at biomarker quartiles, respectively. Non-linearity was investigated by a Likelihood-ratio test (LRT), comparing overall fit between models with the transformed and linear strain CoV biomarker;
3) assessment of strain CoV incremental predictive value in models, adjusted for pre-existing regional strain abnormalities in the respective vector direction. Abnormal regional strain was defined as segmental strain outside the respective reference range in ≥ 1 segments with preserved overall global strain; [27], [39]
4) Kaplan-Meier survival analysis and the logrank test for trend to assess the unadjusted association between biomarker tertiles and outcomes, comparing the two subgroups, reduced LVEF < 50% and preserved LVEF ≥ 50%. [40]

To correct for multiple testing in the principle Cox proportional hazards regression models, statistical significance was assessed at the Bonferroni-corrected a priori α-level 0.05/15 = 0.0033.

## 3 RESULTS

Of 60,746 UKBB participants with available CMR data, 55,132 (90.8%) with complete and valid strain biomarkers were included **(**Supplemental Fig. 8**).** Overall, 26,305 (47.7%) participants were male with mean age 65.4 ± 7.7 years; 28.7% of participants had hypertension, 6.3% diabetes, 6.1% CAD and 0.7% HF **(**Table 1**).** The median (Q1, Q3) LVEDVi, LVESVi, LVMi and LVEF at group level were within normal limits; 3057 (5.5%) had reduced LVEF < 50% and 52,075 (94.5%) had preserved LVEF ≥ 50%. [27], [39], [40] Median global strain was GLS 18.1 (16.7, 19.5) %, GCS 18.8 (17.3, 20.3) % and GRS 31.2 (27.3, 35.3) %; median strain CoV was CoV_LS*,*_ 0.34 (0.29, 0.393) %, CoV_CS*,*_ 0.17 (0.14, 0.20) % and CoV_RS*,*_ 0.28 (0.24, 0.33) %.

**Table 1:** Baseline clinical and CMR-derived characteristics

	Total Cohort (n = 55,132)
Age (years)	65.4±7.7
Male	26,305 (47.7)
White (vs. Non-White)	53,474 (97.0)
Current smoker	1880 (3.4)
BMI (kg/m^2^)	26.5±4.5
*Cardiovascular risk factors*	
Obesity (BMI ≥ 30.0 kg/m^2^)	10,262 (18.6)
Hypertension	15,822 (28.7)
Dyslipidemia	17,207 (31.2)
Diabetes	3486 (6.3)
*Cardiovascular disease*	
Coronary artery disease (CAD)	3361 (6.1)
Previous myocardial infarction (MI)	1407 (2.6)
Chronic kidney disease (CKD)	4374 (7.9)
Cerebrovascular disease	841 (1.5)
Peripheral vascular disease (PVD)	380 (0.7)
*Valvular heart disease*	
Aortic stenosis (AS)	121 (0.2)
Mitral valve disease	257 (0.5)
*Arrhythmia*	
Atrial fibrillation (AF)	1731 (3.1)
Ventricular arrhythmia (VA)	121 (0.2)
*Cardiomyopathy*	
Arrhythmogenic cardiomyopathy (ACM)	16 (0.03)
Dilated cardiomyopathy (DCM)	31 (0.06)
Hypertrophic cardiomyopathy (HCM)	50 (0.09)
Heart failure (HF)	383 (0.7)
*CMR volumes and mass*	
Left ventricular mass (LVMi. g/m^2^)	44.8 (39.6, 51.2)
Left ventricular end-diastolic volume (LVEDVi, mL/m^2^)	76.5 (68.2, 85.9)
Left ventricular end-systolic volume (LVESVi, mL/m^2^)	30.3 (25.5, 36.0)
Left ventricular mass to EDV ratio (LVMVR, g/mL)	0.58 (0.53, 0.65)
Left ventricular ejection fraction (LVEF, %)	60.0 (56.0, 64.1)
Preserved LVEF (≥ 50%)	52,075 (94.5)
Reduced LVEF (< 50%)	3057 (5.5)
*CMR strain parameters*	
Global longitudinal strain (GLS, %)	18.1 (16.7, 19.5)
Global circumferential strain (GCS, %)	18.8 (17.3, 20.3)
Global radial strain (GRS, %)	31.2 (27.3, 35.3)
LS coefficient of variation (CoV_LS_, %)	0.34 (0.29, 0.39)
CS coefficient of variation (CoV_CS_, %)	0.17 (0.14, 0.20)
RS coefficient of variation (CoV_RS_, %)	0.28 (0.24, 0.33)

Data are in mean ± standard deviation (SD) or median (Q1, Q3) for numerical variables and n (%) for categorical variables unless otherwise stated. *CV* cardiovascular, *CMR* cardiovascular magnetic resonance , *BMI* body mass index.

Table 2 presents the reference ranges for segmental, global strain and strain CoV in the longitudinal, circumferential and radial directions, respectively. Supplemental Fig. 9 summarizes the correlation between strain CoV and recognized CMR markers of CV risk. CoV_LS_ and CoV_CS_ had moderate negative correlation with GLS*,* GCS, GRS and LVEF. CoV_RS_ had weak negative correlation with GLS*,* GCS, GRS and LVEF. CoV_CS_ and CoV_RS_ were very strongly correlated (ρ = 0.90), but not with CoV_LS_ (ρ < 0.15). In comparison to strain CoV, global strain biomarkers, GLS*,* GCS and GRS*,* were more strongly correlated with LVEF, LVMi and LVEDVi, respectively.

**Table 2:** Reference ranges of global, segmental strain and strain CoV in the longitudinal, circumferential and radial directions

*SD* standard deviation, *LL* lower limit, *UL* upper limit, *GLS* global longitudinal strain, *CoV*_*LS*_ longitudinal strain coefficient of variation, *LS* longitudinal strain, *AHA* American Heart Association, *GCS* global circumferential strain, *CoV*_*CS*_ circumferential strain coefficient of variation, *GRS* global radial strain, *CoV*_*CS*_ radial strain coefficient of variation.

### 3.1 Associations with prevalent CV risk and disease

**Fig. 2:**
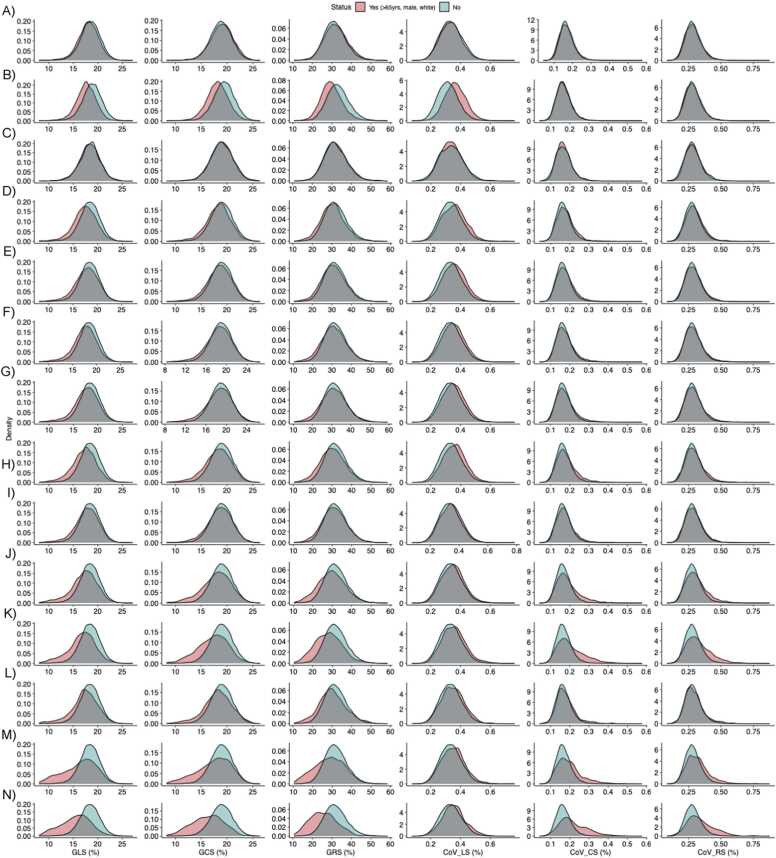
**Probability density charts of global strain and strain CoV by CV risk factors and disease.** A) Age >65 years, B) Male sex, C) White ethnicity, D) Current smoker, E) Obesity, F) Dyslipidemia, G) Hypertension, H) Diabetes mellitus, I) Chronic kidney disease, J) Coronary artery disease, K) Previous myocardial infarction, L) Stroke, M) Atrial fibrillation and N) Heart failure. *CoV* coefficient of variation, *CV* cardiovascular

The healthy reference cohort included 24,026 (43.6%), of whom 9575 (39.9%) were male with mean age 63.4 ± 7.4 years. Table 3 and Fig. 2 present the median (Q1, Q3) and distribution of global strain and strain CoV, stratified by age, sex and ethnicity in the healthy reference cohort as well as by CV risk factors and disease status, respectively**.** Participants > 65 years of age had lower CoV in the 3 strain vector directions and lower GLS, but greater GCS and GRS versus participants ≤ 65 years of age (P < 0.05 for all). Male sex was associated with higher CoV_LS_, CoV_CS*,*_ CoV_RS_ and lower GLS, GCS*,* GRS (P < 0.001 for all). Generally, higher CoV_LS_, CoV_CS_, CoV_RS_ and lower GLS, GCS*,* GRS were associated with prevalent CV risk factors (i.e. smoking, obesity, dyslipidemia, hypertension and diabetes) and disease (i.e. CAD, previous MI, stroke, AF and HF), respectively (P < 0.05 for all). Participants with prevalent HF had the greatest heterogeneity in segmental strain and lowest global strain in the longitudinal, circumferential and radial directions (P < 0.001 for all, Table 3**).** There was no association between CoV_RS_ and stroke (P > 0.05).

**Table 3:** Strain biomarkers, stratified by age group, sex, ethnicity, CV risk and disease

	GLS (%)	GCS (%)	GRS (%)	CoV_LS_ (%)	CoV_CS_ (%)	CoV_RS_ (%)
Healthy reference	18.4 (17.0, 19.7)	18.9 (17.5, 20.3)	31.4 (27.8, 35.4)	0.33 (0.28, 0.38)	0.16 (0.14, 0.19)	0.28 (0.24, 0.32)
Age						
> 65 years	18.2 (16.8, 19.5)†	19.0 (17.6, 20.5)†	31.8 (27.9, 35.9)†	0.33 (0.28, 0.38)†	0.16 (0.14, 0.19)†	0.28 (0.24, 0.32)*
≤ 65 years	18.5 (17.2, 19.8)†	18.8 (17.5, 20.2)†	31.2 (27.7, 35.1)†	0.34 (0.29, 0.39)†	0.17 (0.14, 0.19)†	0.28 (0.25, 0.32)*
Sex						
Male	17.7 (16.4, 18.9)†	18.2 (16.8, 19.5)†	29.4 (26.2, 33.§)†	0.36 (0.32, 0.41)†	0.17 (0.14, 0.19)†	0.28 (0.24, 0.32)†
Female	18.8 (17.5, 20.1)†	19.4 (18.1, 20.7)†	32.8 (29.1, 36.6)†	0.31 (0.27, 0.36)†	0.16 (0.14, 0.19)†	0.28 (0.24, 0.33)†
Ethnicity						
White	18.4 (17.0, 19.7)	18.9 (17.5, 20.3)	31.4 (27.8, 35.4)	0.33 (0.28, 0.38)	0.16 (0.14, 0.19)	0.28 (0.24, 0.32)
Non-white	18.5 (17.2, 19.6)	19.0 (17.6, 20.5)	31.4 (28.2, 36.0)	0.34 (0.28, 0.39)	0.16 (0.14, 0.19)	0.28 (0.24, 0.32)
Smoker	17.5 (16.0, 19.0)†	18.5 (16.9, 19.9)†	30.3 (26.4, 34.1)†	0.35 (0.30, 0.41)†	0.17 (0.15, 0.20)†	0.29 (0.25, 0.33)†
Obesity	17.9 (16.3, 19.5)†	18.6 (17.0, 20.1)†	30.6 (26.6, 34.7)†	0.35 (0.30, 0.41)†	0.17 (0.14, 0.20)†	0.29 (0.24, 0.33)†
Hypercholesterolemia	17.7 (16.2, 19.2)†	18.7 (17.0, 20.2)†	30.8 (26.7, 35.1)†	0.35 (0.30, 0.40)†	0.17 (0.14, 0.20)†	0.28 (0.24, 0.33)†
Hypertension	17.8 (16.2, 19.3)†	18.8 (17.0, 20.3)†	31.1 (26.8, 35.5)†	0.35 (0.30, 0.40)†	0.17 (0.14, 0.20)†	0.28 (0.24, 0.33)†
Diabetes mellitus	17.4 (15.8, 18.9)†	18.3 (16.6, 19.9)†	30.0 (25.8, 34.3)†	0.36 (0.31, 0.41)†	0.17 (0.15, 0.21)†	0.29 (0.25, 0.34)†
Chronic kidney disease	17.9 (16.4, 19.4)†	18.7 (17.1, 20.2)†	31.0 (26.9, 35.2)†	0.34 (0.29, 0.39)†	0.17 (0.14, 0.20)†	0.28 (0.24, 0.33)*
Coronary artery disease	17.4 (15.7, 19.0)†	18.2 (16.2, 19.9)†	29.7 (24.9, 34.3)†	0.35 (0.30, 0.40)†	0.18 (0.15, 0.22)†	0.30 (0.25, 0.35)†
Previous MI	16.9 (15.2, 18.6)†	17.6 (15.3, 19.4)†	28.1 (23.1, 32.8)†	0.35 (0.30, 0.40)†	0.19 (0.15, 0.24)†	0.31 (0.26, 0.38)†
Cerebrovascular disease	17.4 (15.9, 19.0)†	18.3 (16.6, 20.0)†	29.9 (25.8, 34.4)†	0.34 (0.30, 0.40)†	0.17 (0.15, 0.20)†	0.28 (0.24, 0.33)
Atrial fibrillation	16.8 (14.1, 18.8)†	17.9 (15.4, 20.0)†	28.8 (23.2, 34.3)†	0.35 (0.30, 0.40)†	0.18 (0.15, 0.23)†	0.30 (0.25, 0.36)†
Heart failure	15.7 (13.5, 17.6)†	16.5 (14.2, 18.3)†	25.2 (20.8, 30.0)†	0.35 (0.31, 0.40)†	0.20 (0.17, 0.26)†	0.32 (0.27, 0.40)†

Data are presented as median (Q1, Q3). *GLS* global longitudinal strain, *GCS* global circumferential strain, *GRS* global radial strain, *CoV*_*LS*_ longitudinal strain coefficient of variation, *CoV*_*CS*_ circumferential strain coefficient of variation, *CoV*_*RS*_ radial strain coefficient of variation, *MI* myocardial infarction. *P < 0.05. †P < 0.001.

### 3.2 Strain models of incident cardiac disease

Over a median follow-up of 5.1 (2.2, 6.5) years since the baseline CMR exam, the composite CV endpoint occurred in 1537/55,132 (2.8%) participants; this included 490 (0.9%) MI, 410 HF diagnoses (0.7%) and 974 (1.8%) arrhythmia events. Death occurred in 723 (1.3%) participants.

#### 3.2.1 Biomarker predictive strength

Supplemental Tables 3–7 summarize biomarker HR (95% CI) and performance measures from **Models 1** to **3** for the composite CV endpoint, MI, HF, arrhythmia and death from any cause, respectively. We summarized the important strain findings from the fully adjusted multivariable clinical CMR **Model 3.**

**Fig. 3:**
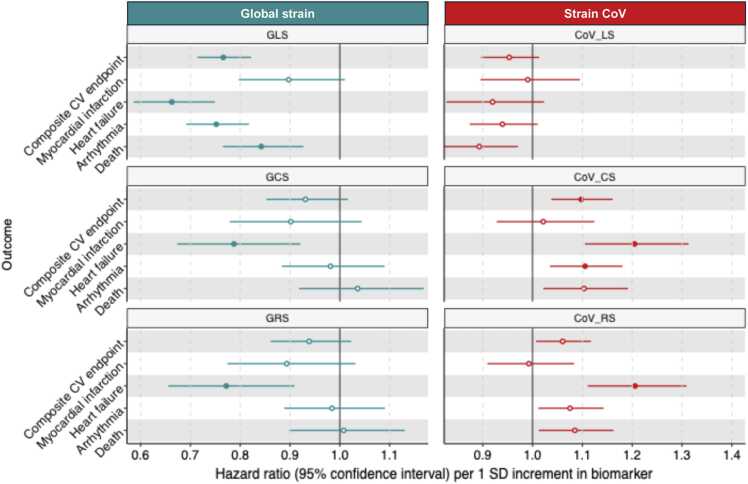
**Forest plots, comparing CoV and global strain to predict CV endpoints.** CoV_CS_*,* CoV_RS_ and GLS were predictive of death from any cause and a composite CV endpoint (MI, HF and arrhythmia) after adjusting for clinical and cardiovascular magnetic resonance imaging risk markers for CV disease. Correction for multiple testing rendered the association between CoV_RS_*,* CoV_CS_*,* and death from any cause non-significant as well as between CoV_RS_*,* HF and arrhythmia non-significant. GCS and GRS were associated with HF. *Filled vs. unfilled marker indicates Bonferroni-corrected P < 0.0033 vs. > 0.0033. *CoV* coefficient of variation, *GLS* global longitudinal strain, *CoV*_*LS*_ longitudinal strain coefficient of variation, *CV* cardiovascular, *GCS* global circumferential strain, *CoV*_*CS*_ circumferential strain coefficient of variation, *GRS* global radial strain, *CoV*_*RS*_ radial strain coefficient of variation, *MI* myocardial infarction, *HF* heart failure.

For longitudinal strain**,** 1 SD decrease in GLS was associated with 23% (HR 0.77 [0.71–0.82]), 34% (HR 0.66 [0.59–0.75]), 25% (HR 0.75 [0.69–0.82]) and 16% (HR 0.84 [0.77–0.93]) greater risk of the composite CV endpoint, HF, arrhythmia and death from any cause, respectively **(**Fig. 3 and Table 4). By comparison, CoV_LS_ was not associated with any outcome after applying correction for multiple testing (P > 0.05 for all).

**Table 4:** Results and performance measures of Cox proportional hazards regression Model 3 to predict study endpoints

Outcome	Biomarker		HR	CI	P-value*	△C-index	C-index	P-value†	NRI(>0) [%]	△ Brier score	△AIC	△χ^2^	P-value‡
Composite CV endpoint	CoV_LS_		0.95	0.90 - 1.01	1.00	−0.001	0.728	0.16	0.0 (−2.4 - 3.8)	0.00000	0	−2.4	0.12
	GLS		0.77	0.71 - 0.82	<0.001	-	-	-	-	-	-	-	-
	CoV_CS_		1.10	1.04 - 1.16	0.017	0.001	0.729	<0.001	2.3 (−0.9 - 5.3)	0.00001	−8	−10.4	0.001
	GCS		0.93	0.85 - 1.02	1.00	-	-	-	-	-	-	-	-
	CoV_RS_		1.06	1.01 - 1.12	0.40	0.001	0.728	0.80	2.0 (−1.7 - 4.6)	0.00001	−3	−4.8	0.028
	GRS		0.94	0.86 - 1.02	1.00	-	-	-	-	-	-	-	-
Myocardial infarction	CoV_LS_		0.99	0.90 - 1.10	1.00	0.000	0.730	0.46	−2 (−3.7 - 7.4)	0.00000	2	0.0	0.85
	GLS		0.90	0.80 - 1.01	1.00	-	-	-	-	-	-	-	-
	CoV_CS_		1.02	0.93 - 1.12	1.00	0.000	0.731	0.057	−0.4 (−3.8 - 5.7)	0.00000	2	−0.2	0.67
	GCS		0.90	0.78 - 1.04	1.00	-	-	-	-	-	-	-	-
	CoV_RS_		0.99	0.91 - 1.08	1.00	0.000	0.730	0.24	1.2 (−3.1 - 6.1)	0.00000	2	0.0	0.87
	GRS		0.89	0.77 - 1.03	1.00	-	-	-	-	-	-	-	-
Heart failure	CoV_LS_		0.92	0.83 - 1.02	1.00	0.001	0.824	0.24	−2.7 (−12 - 7.2)	0.00000	0	−2.4	0.12
	GLS		0.66	0.59 - 0.75	<0.001	-	-	-	-	-	-	-	-
	CoV_CS_		1.20	1.11 - 1.31	<0.001	0.004	0.826	<0.001	1.5 (−4.6 - 7.7)	0.00002	−15	−17.1	<0.001
	GCS		0.79	0.67 - 0.92	0.041	-	-	-	-	-	-	-	-
	CoV_RS_		1.21	1.11 - 1.31	<0.001	0.004	0.825	<0.001	5.2 (−1.3 - 10.8)	0.00002	−17	−18.9	<0.001
	GRS		0.77	0.66 - 0.91	0.028	-	-	-	-	-	-	-	-
Arrhythmia	CoV_LS_		0.94	0.87 - 1.01	1.38	0.000	0.736	0.30	1.6 (−2.2 - 5.3)	0.00000	−1	−2.8	0.092
	GLS		0.75	0.69 - 0.82	<0.001	-	-	-	-	-	-	-	-
	CoV_CS_		1.11	1.03 - 1.18	0.044	0.002	0.736	0.001	2.9 (−0.7 - 6.2)	0.00001	−7	−8.6	0.003
	GCS		0.98	0.88 - 1.09	1.00	-	-	-	-	-	-	-	-
	CoV_RS_		1.07	1.01 - 1.14	0.30	0.001	0.735	0.004	2.6 (−1.0 - 6.2)	0.00001	−3	−5.3	0.021
	GRS		0.98	0.89 - 1.09	1.00	-	-	-	-	-	-	-	-
Death from any cause	CoV_LS_		0.89	0.82 - 0.97	0.12	0.001	0.728	0.50	0.4 (−3.6 - 4.4)	0.00000	−5	−7.1	0.008
	GLS		0.84	0.77 - 0.93	0.007	-	-	-	-	-	-	-	-
	CoV_CS_		1.10	1.02 - 1.19	0.18	−0.001	0.724	0.31	2.8 (−1.7 - 7.1)	0.00000	−4	−6.1	0.013
	GCS		1.04	0.92 - 1.17	1.00	-	-	-	-	-	-	-	-
	CoV_RS_		1.08	1.01 - 1.16	0.31	0.000	0.725	0.065	1.5 (−2.4 - 5.8)	0.00000	−3	−5.2	0.022
	GRS		1.01	0.90 - 1.13	1.00	-	-	-	-	-	-	-	-

*HR* hazard ratio, *CI* confidence interval, *NRI(>0)* continuous net reclassification improvement, *AIC* Akaike information criterion, *CV* cardiovascular, *CoV*_*LS*_ longitudinal strain coefficient of variation, *GLS* global longitudinal strain, *CoV*_*CS*_ circumferential strain coefficient of variation, *GCS* global circumferential strain, *CoV*_*RS*_ radial strain coefficient of variation, *GRS* global radial strain. *Multiplied by Bonferroni correction factor 15. †P-value, comparing △C-index between boot-strapped samples. ‡P-value, comparing chi-square (χ^2^) estimates of nested models by the Likelihood ratio test.

For circumferential and radial strain, 1 SD decrease in GCS and GRS was associated with 21% (HR 0.79 [0.67–0.92]) and 23% (HR 0.77 [0.66–0.91]) greater risk of HF, respectively **(**Fig. 3 and Table 4). In contrast, 1 SD increase in CoV_CS_ and CoV_RS_ was associated with 10% (HR 1.10 [1.04–1.16]) and 6% (HR 1.06 [1.01–1.12]) greater risk of the composite CV endpoint; 20% (HR 1.20 [1.11–1.31]) and 21% (HR 1.21 [1.11–1.31) greater risk of HF; 11% (HR 1.11 [1.03–1.18]) and 7% (HR 1.07 [1.01–1.14]) greater risk of arrhythmia; and finally, 10% (HR 1.10 [1.02–1.19]) and 8% (HR 1.08 [1.01–1.16]) greater risk of death from any cause, respectively **(**Fig. 3 and Table 4). Multiple testing correction rendered CoV_CS_’s association with mortality and CoV_RS_’s associations with the composite CV endpoint, arrhythmia and mortality non-significant (P > 0.05 for all).

Both CoV and global strain were not associated with incident MI (P > 0.05 for all).

#### 3.2.2 Added prognostic value of biomarker

In the fully adjusted multivariable clinical CMR **Model 3**, CoV_CS_ significantly improved the discrimination of the composite CV endpoint (△C-index 0.001 [P < 0.001], Table 4**).** Both CoV_CS_ and CoV_RS*,*_ provided incremental prognostic value above LVMi, LVEDVi, LVEF and their reciprocal global strain marker in stratifying participant risk of incident HF (△C-index 0.004 [P < 0.001] for both, respectively) and incident arrhythmia (△C-index 0.002 [P = 0.001] and 0.001 [P = 0.004], respectively, Table 4**)**. However, CoV_LS_*,* CoV_CS_ and CoV_RS_ did not improve **Model 3’s** discrimination of incident MI or death from any cause (△C-index P-value > 0.05 for all, respectively).

The addition of CoV_CS_ and CoV_RS_ to **Model 3** led to non-significant improvements in event risk reclassification for the composite CV endpoint, HF, arrhythmia and death from any cause **(**Table 4**)**. Average NRI(>0) ranged from 1.5% to 5.2%.

#### 3.2.3 Overall model performance

For **Models 1** to **3,** Brier scores at 5 years changed minimally, suggesting that CoV did not improve model predictive accuracy. However, lower AIC by > 2 and statistically significant improvements in the likelihood functions (P < 0.05) after the addition of CoV_CS_ and CoV_RS*,*_ indicated overall improvement in performance across all models for endpoints, except incident MI **(**Table 4 and Supplemental Tables 3–7**)**. This was not consistently seen with CoV_LS_*.*

### 3.3 Sensitivity analyses

Overall, the described associations between strain markers and outcomes were maintained in groups, stratified by age and sex **(**Table 5**).** However, the independent predictive value of CoV_CS_ and CoV_RS_ for the composite CV endpoint, HF and arrhythmia increased among participants > 65 years of age and female (P < 0.05 for all**)**, whereas that of GLS increased among participants ≤ 65 years of age and male (P < 0.05 for all**)**. The association between lower GLS*,* CoV_LS_ greater CoV_CS_*,* CoV_RS_ and death from any cause was driven by their association among participants > 65 years of age and male, respectively (P < 0.05 for all).

**Table 5:** Results of Cox proportional hazards regression Model 3 to predict study endpoints, stratified by age and sex

Outcome	Biomarker	HR	CI	P-value	HR	CI	P-value
		Age > 65 years			Age ≤ 65 years		
Composite CV endpoint	CoV_LS_	0.96	0.9 - 1.03	0.30	0.93	0.82 - 1.04	0.21
	GLS	0.78	0.72 - 0.85	<0.001	0.72	0.63 - 0.83	<0.001
	CoV_CS_	1.13	1.05 - 1.21	0.001	1.05	0.95 - 1.17	0.35
	GCS	0.98	0.89 - 1.10	0.78	0.81	0.69 - 0.95	0.010
	CoV_RS_	1.08	1.02 - 1.15	0.010	1.01	0.92 - 1.12	0.78
	GRS	0.98	0.89 - 1.09	0.73	0.83	0.70 - 0.98	0.025
Myocardial infarction	CoV_LS_	0.97	0.86 - 1.09	0.57	1.05	0.88 - 1.26	0.56
	GLS	0.86	0.75 - 1.00	0.045	0.99	0.80 - 1.22	0.91
	CoV_CS_	1.04	0.92 - 1.17	0.55	1.00	0.85 - 1.18	0.97
	GCS	0.95	0.79 - 1.14	0.56	0.82	0.64 - 1.05	0.12
	CoV_RS_	1.01	0.90 - 1.12	0.89	0.97	0.83 - 1.13	0.71
	GRS	0.94	0.79 - 1.13	0.53	0.80	0.63 - 1.02	0.073
Heart failure	CoV_LS_	0.92	0.82 - 1.03	0.15	0.89	0.70 - 1.13	0.35
	GLS	0.69	0.60 - 0.79	<0.001	0.56	0.43 - 0.74	<0.001
	CoV_CS_	1.25	1.13 - 1.38	<0.001	1.11	0.92 - 1.34	0.27
	GCS	0.87	0.73 - 1.04	0.13	0.53	0.38 - 0.74	<0.001
	CoV_RS_	1.24	1.13 - 1.37	<0.001	1.12	0.92 - 1.35	0.26
	GRS	0.83	0.69 - 1.00	0.056	0.56	0.39 - 0.82	0.003
Arrhythmia	CoV_LS_	0.96	0.89 - 1.04	0.34	0.87	0.74 - 1.01	0.073
	GLS	0.77	0.70 - 0.85	<0.001	0.68	0.57 - 0.81	<0.001
	CoV_CS_	1.14	1.06 - 1.24	0.001	1.01	0.88 - 1.17	0.86
	GCS	1.01	0.89 - 1.15	0.86	0.89	0.72 - 1.10	0.29
	CoV_RS_	1.11	1.03 - 1.19	0.005	0.99	0.86 - 1.13	0.84
	GRS	1.00	0.89 - 1.13	0.99	0.93	0.75 - 1.16	0.53
Death from any cause	CoV_LS_	0.87	0.80 - 0.96	0.005	0.97	0.81 - 1.16	0.75
	GLS	0.83	0.75 - 0.93	0.001	0.87	0.71 - 1.07	0.18
	CoV_CS_	1.13	1.03 - 1.24	0.007	1.03	0.88 - 1.21	0.70
	GCS	1.10	0.96 - 1.27	0.18	0.84	0.66 - 1.07	0.17
	CoV_RS_	1.11	1.03 - 1.20	0.010	1.01	0.87 - 1.18	0.85
	GRS	1.07	0.93 - 1.22	0.35	0.84	0.66 - 1.06	0.13
		Male			Female		
Composite CV endpoint	CoV_LS_	0.92	0.86 - 0.99	0.029	1.02	0.92 - 1.13	0.72
	GLS	0.75	0.69 - 0.82	<0.001	0.84	0.75 - 0.94	0.003
	CoV_CS_	1.07	1.00 - 1.15	0.047	1.14	1.03 - 1.25	0.007
	GCS	0.91	0.81 - 1.01	0.071	1.01	0.88 - 1.16	0.88
	CoV_RS_	1.04	0.97 - 1.11	0.24	1.09	1.00 - 1.19	0.042
	GRS	0.92	0.83 - 1.02	0.11	1.00	0.88 - 1.15	0.97
Myocardial infarction	CoV_LS_	0.93	0.83 - 1.04	0.19	1.18	0.98 - 1.41	0.078
	GLS	0.85	0.74 - 0.98	0.021	1.06	0.86 - 1.31	0.61
	CoV_CS_	1.08	0.96 - 1.21	0.18	0.90	0.75 - 1.08	0.26
	GCS	0.93	0.79 - 1.11	0.43	0.84	0.65 - 1.09	0.19
	CoV_RS_	1.04	0.94 - 1.16	0.45	0.89	0.75 - 1.05	0.17
	GRS	0.91	0.77 - 1.08	0.28	0.87	0.68 - 1.11	0.26
Heart failure	CoV_LS_	0.85	0.75 - 0.96	0.010	1.11	0.93 - 1.34	0.26
	GLS	0.62	0.54 - 0.72	<0.001	0.82	0.66 - 1.01	0.061
	CoV_CS_	1.19	1.07 - 1.32	0.002	1.26	1.08 - 1.48	0.003
	GCS	0.76	0.63 - 0.92	0.005	0.90	0.69 - 1.17	0.44
	CoV_RS_	1.18	1.07 - 1.31	0.001	1.28	1.10 - 1.48	0.001
	GRS	0.74	0.61 - 0.90	0.003	0.89	0.68 - 1.16	0.38
Arrhythmia	CoV_LS_	0.91	0.84 - 0.99	0.031	1.01	0.89 - 1.14	0.91
	GLS	0.74	0.67 - 0.82	<0.001	0.82	0.72 - 0.94	0.005
	CoV_CS_	1.07	0.98 - 1.16	0.13	1.19	1.06 - 1.33	0.003
	GCS	0.93	0.82 - 1.06	0.26	1.12	0.94 - 1.32	0.20
	CoV_RS_	1.04	0.97 - 1.13	0.28	1.14	1.02 - 1.26	0.016
	GRS	0.94	0.83 - 1.06	0.33	1.08	0.92 - 1.26	0.35
Death from any cause	CoV_LS_	0.90	0.81 - 0.99	0.030	0.91	0.79 - 1.04	0.16
	GLS	0.81	0.72 - 0.91	<0.001	0.91	0.78 - 1.07	0.26
	CoV_CS_	1.14	1.04 - 1.25	0.008	1.04	0.91 - 1.18	0.60
	GCS	1.02	0.88 - 1.18	0.81	1.06	0.88 - 1.28	0.51
	CoV_RS_	1.11	1.02 - 1.21	0.017	1.04	0.92 - 1.17	0.52
	GRS	0.98	0.85 - 1.13	0.75	1.07	0.89 - 1.28	0.46

*HR* hazard ratio, *CI* confidence interval, *CV* cardiovascular, *CoV*_*LS*_ longitudinal strain coefficient of variation, *CoV*_*CS*_ circumferential strain coefficient of variation, *CoV*_*RS*_ radial strain coefficient of variation, *GLS* global longitudinal strain,*GCS* global circumferential strain, *GRS* global radial strain.

Comparisons were drawn between the overall cohort and the following subgroups. There was no association between global strain, strain CoV and the outcomes, HF and death from any cause among participants with prevalent MI **(**Table 6**).** Participants with prevalent CKD had stronger associations between lower GLS*,* GCS*,* GRS*,* CoV_LS_, greater CoV_RS_ and HF, respectively; and between lower GLS*,* greater CoV_CS_*,* CoV_RS_ and death from any cause, respectively (P < 0.05 for all, Table 6**).** Participants with prevalent AF had stronger associations between lower GLS*,* CoV_LS_, greater CoV_CS_ and HF (P < 0.05 for all, Table 6**).** The strength of association between higher CoV_CS_, CoV_RS_ and HF increased among participants with preserved LVEF ≥ 50% and ≥ 3 risk factors for HFpEF **(**HR 1.37 [1.14–1.65]; HR1.35 [1.14–1.60, respectively; Table 6**).** This was not observed with lower GLS **(**HR 0.68 [0.53–0.86])**.** There was no association between strain markers and death from any cause in the subgroups with prevalent AF and at increased risk of HFpEF, respectively (P > 0.05 for all).

**Table 6:** Model 3 sensitivity analyses in predefined subgroups to predict heart failure and death from any cause

Outcome		Global strain	HR	CI	P-value		Strain CoV	HR	CI	P-value
*Prevalent myocardial infarction*										
Heart failure		GLS	0.72	0.46 - 1.12	0.14		CoV_LS_	0.81	0.60 - 1.11	0.20
		GCS	0.81	0.47 - 1.41	0.45		CoV_CS_	1.22	0.89 - 1.67	0.21
		GRS	0.74	0.42 - 1.30	0.29		CoV_RS_	1.23	0.92 - 1.65	0.16
Death from any cause		GLS	1.18	0.76 - 1.84	0.46		CoV_LS_	0.81	0.58 - 1.14	0.23
		GCS	1.64	0.88 - 3.06	0.12		CoV_CS_	1.26	0.87 - 1.81	0.22
		GRS	1.53	0.86 - 2.72	0.15		CoV_RS_	1.32	0.95 - 1.82	0.10
*Prevalent chronic kidney disease*										
Heart failure		GLS	0.41	0.31 - 0.54	<0.001		CoV_LS_	0.70	0.53 - 0.92	0.011
		GCS	0.59	0.42 - 0.84	0.003		CoV_CS_	1.24	0.99 - 1.56	0.055
		GRS	0.54	0.37 - 0.78	0.001		CoV_RS_	1.26	1.02 - 1.56	0.032
Death from any cause		GLS	0.70	0.53 - 0.93	0.015		CoV_LS_	0.88	0.69 - 1.12	0.30
		GCS	1.11	0.76 - 1.64	0.58		CoV_CS_	1.26	1.01 - 1.56	0.038
		GRS	1.05	0.73 - 1.52	0.80		CoV_RS_	1.24	1.02 - 1.50	0.028
*Prevalent atrial fibrillation*										
Heart failure		GLS	0.56	0.36 - 0.87	0.009		CoV_LS_	0.64	0.47 - 0.89	0.007
		GCS	1.05	0.61 - 1.81	0.85		CoV_CS_	1.36	1.02 - 1.83	0.039
		GRS	0.99	0.58 - 1.69	0.96		CoV_RS_	1.24	0.96 - 1.62	0.10
Death from any cause		GLS	0.94	0.60 - 1.48	0.79		CoV_LS_	1.00	0.71 - 1.42	0.98
		GCS	1.14	0.63 - 2.07	0.66		CoV_CS_	1.30	0.91 - 1.84	0.15
		GRS	1.02	0.58 - 1.79	0.95		CoV_RS_	1.17	0.88 - 1.57	0.29
*Preserved LVEF & ≥ 3 HFpEF risk factors (age > 65 years, obesity, hypertension, diabetes, CKD and/or AF)*										
Heart failure		GLS	0.68	0.53 - 0.86	0.001		CoV_LS_	0.89	0.71 - 1.10	0.26
		GCS	0.96	0.71 - 1.30	0.81		CoV_CS_	1.37	1.14 - 1.65	0.001
		GRS	0.91	0.67 - 1.23	0.54		CoV_RS_	1.35	1.14 - 1.60	0.001
Death from any cause		GLS	0.88	0.70 - 1.09	0.25		CoV_LS_	0.87	0.72 - 1.05	0.15
		GCS	1.00	0.76 - 1.32	0.98		CoV_CS_	1.04	0.86 - 1.26	0.70
		GRS	0.98	0.75 - 1.27	0.87		CoV_RS_	1.01	0.85 - 1.20	0.87

*HR* hazard ratio, *CI* confidence interval, *CoV* coefficient of variation, *GLS* global longitudinal strain, *GCS* global circumferential strain, *GRS* global radial strain, *CoV*_*LS*_ longitudinal strain coefficient of variation, *CoV*_*CS*_ circumferential strain coefficient of variation, *CoV*_*RS*_ radial strain coefficient of variation, *HFpEF* heart failure with preserved ejection fraction, *CKD* chronic kidney disease, *AF* atrial fibrillation.

### 3.4 Non-linear effect of strain CoV

**Fig. 4:**
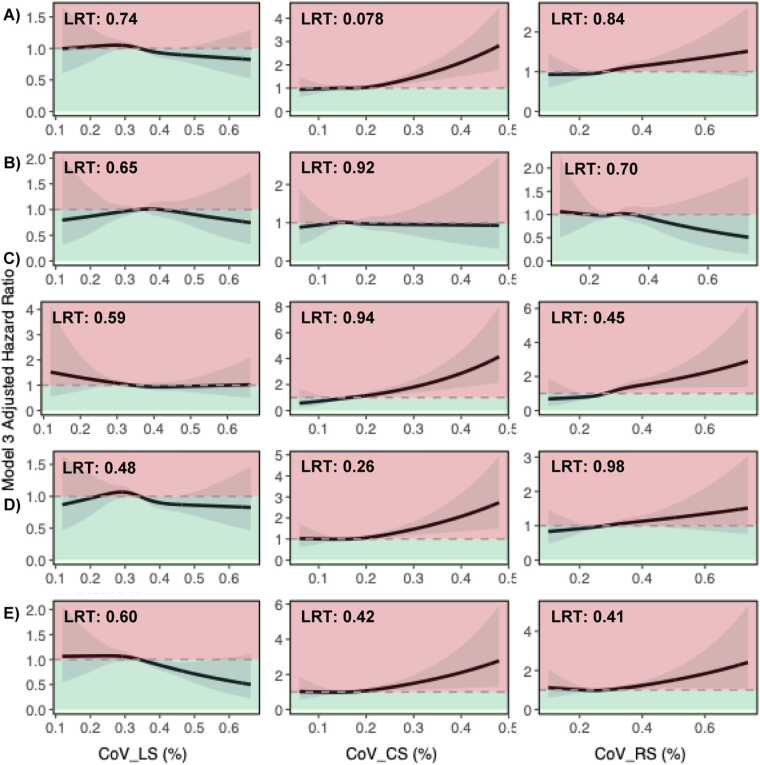
**RCS plots, assessing the non-linear relationship between strain CoV and the outcomes A) composite CV endpoint, B) MI, C) HF, D) arrhythmia and E) death from any cause.** There was no graphical or statistical evidence to support a plausible non-linear relationship between strain CoV_LS_*,* CoV_CS_*,* CoV_RS_ and predefined study endpoints (P > 0.05 for all). *LRT* Likelihood-ratio test, *RCS* restricted cubic splines, *CoV* coefficient of variation, *CV* cardiovascular, *MI* myocardial infarction, *HF* heart failure, *CoV*_*LS*_ longitudinal strain coefficient of variation, *CoV*_*CS*_ circumferential strain coefficient of variation, *CoV*_*RS*_ radial strain coefficient of variation.

There was no consistent evidence from RCS plots and Likelihood ratio tests that the relationship between CoV_LS_*,* CoV_CS_*,* CoV_RS_ and outcomes was non-linear (P > 0.05 for all, Fig. 4).

### 3.5 Interaction between strain CoV & regional dysfunction

From 55,132 participants, 17,962 (32.6%), 17,813 (32.3%) and 17,871 (32.4%) had abnormal regional strain measured in the longitudinal, circumferential and radial directions, respectively. In **Model 3**, adjusted for presence of regional strain abnormalities, CoV_CS_ and CoV_RS_ added independent incremental predictive value in the discrimination of the composite CV endpoint (△C-index 0.001 [P < 0.05] for both, respectively), HF (△C-index 0.004 [P < 0.001] for both, respectively) and arrhythmia (△C-index 0.002 [P = 0.001] and 0.001 [P = 0.017], respectively) **(**Table 7**).**

**Table 7:** Results of Cox proportional hazards regression Model 3, adjusted for strain CoV and regional strain abnormalities

Outcome	Biomarker	HR	CI	P-value	△C-index	C-index	P-value	NRI (>0) [%]	△ Brier score	△AIC
Composite CV endpoint	CoV_LS_	1.02	0.96 - 1.08	0.47	0.000	0.729	0.78	0.9 (−3.3 - 4.9)	0.00000	1
	LS regionality	1.26	1.12 - 1.41	<0.001	-	-	-	-	-	-
	CoV_CS_	1.10	1.05 - 1.16	<0.001	0.001	0.729	<0.001	1.6 (−1.2 - 4.6)	0.00000	−11
	CS regionality	1.11	0.99 - 1.25	0.071	-	-	-	-	-	-
	CoV_RS_	1.06	1.01 - 1.12	0.026	0.001	0.729	0.007	2.0 (−0.9 - 4.4)	0.00001	−3
	RS regionality	1.14	1.01 - 1.28	0.028	-	-	-	-	-	-
Myocardial infarction	CoV_LS_	1.02	0.92 - 1.12	0.73	0.001	0.731	0.18	1.2 (−3.4 - 6.1)	0.00000	2
	LS regionality	1.13	0.93 - 1.37	0.20	-	-	-	-	-	-
	CoV_CS_	1.05	0.96 - 1.15	0.33	0.001	0.731	0.21	0.4 (−3.5 - 6.8)	0.00000	1
	CS regionality	0.98	0.81 - 1.19	0.83	-	-	-	-	-	-
	CoV_RS_	1.00	0.92 - 1.09	0.98	0.000	0.730	0.23	0.2 (−2.6 - 5.5)	0.00000	2
	RS regionality	0.99	0.82 - 1.20	0.95	-	-	-	-	-	-
Heart failure	CoV_LS_	1.02	0.92 - 1.13	0.69	0.000	0.822	0.43	4.8 (−6.1 - 11.6)	0.00000	2
	LS regionality	1.45	1.16 - 1.80	0.001	-	-	-	-	-	-
	CoV_CS_	1.24	1.15 - 1.35	<0.001	0.004	0.825	<0.001	3.1 (−2.8 - 8.0)	0.00001	−24
	CS regionality	1.26	1.01 - 1.57	0.042	-	-	-	-	-	-
	CoV_RS_	1.21	1.12 - 1.31	<0.001	0.004	0.826	<0.001	4.7 (−1.5 - 10.4)	0.00001	−18
	RS regionality	1.30	1.05 - 1.62	0.016	-	-	-	-	-	-
Arrhythmia	CoV_LS_	1.01	0.95 - 1.08	0.72	0.000	0.737	0.17	1.1 (−2.6 - 4.5)	0.00000	2
	LS regionality	1.27	1.11 - 1.46	0.001	-	-	-	-	-	-
	CoV_CS_	1.10	1.03 - 1.17	0.004	0.002	0.736	0.001	1.5 (−2.6 - 4.9)	0.00001	−6
	CS regionality	1.12	0.98 - 1.29	0.11	-	-	-	-	-	-
	CoV_RS_	1.07	1.01 - 1.14	0.028	0.001	0.736	0.017	2.4 (−1 - 6.2)	0.00001	−3
	RS regionality	1.12	0.98 - 1.29	0.095	-	-	-	-	-	-
Death from any cause	CoV_LS_	0.94	0.87 - 1.01	0.11	0.000	0.725	0.74	0.6 (−3.0 - 5.1)	0.00000	−1
	LS regionality	1.04	0.89 - 1.22	0.60	-	-	-	-	-	-
	CoV_CS_	1.09	1.02 - 1.17	0.016	0.000	0.724	0.96	1.0 (−2.6 - 5.4)	0.00000	−4
	CS regionality	1.05	0.90 - 1.23	0.55	-	-	-	-	-	-
	CoV_RS_	1.08	1.01 - 1.16	0.026	0.000	0.725	0.079	1.7 (−2.5 - 5.8)	0.00000	−3
	RS regionality	1.05	0.90 - 1.23	0.53	-	-	-	-	-	-

*CoV* coefficient of variation, *HR* hazard ratio, *CI*: confidence interval, *NRI(>0)* continuous net reclassification improvement, *AIC* Akaike information criterion, *CV* cardiovascular, *CoV*_*LS*_ longitudinal strain coefficient of variation, *LS* longitudinal strain, *CoV*_*CS*_ circumferential strain coefficient of variation, *CS* circumferential strain, *CoV*_*RS*_ radial strain coefficient of variation, *RS* radial strain.

### 3.6 Kaplan-Meier cumulative hazards by strain tertiles and LVEF

**Fig. 5:**
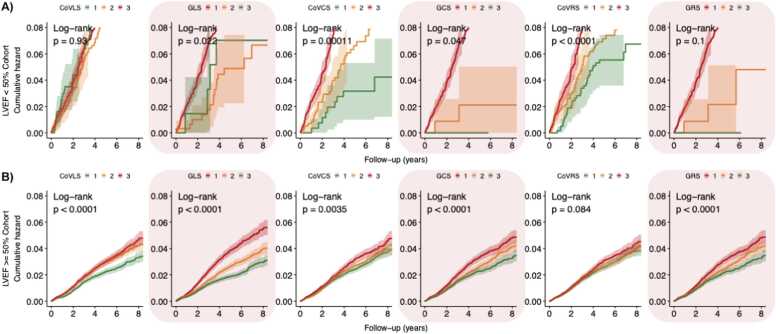
**Strain biomarker KM hazard curves by tertiles for the composite CV endpoint.** In participants with reduced LVEF < 50% **(panel A),** CoV_CS_ and CoV_RS_ stratified the risk of the composite CV endpoint better than global strain. Conversely, in participants with preserved LVEF ≥ 50% **(Panel B),** GLS*,* GCS and GRS were more sensitive markers for increased risk of the composite CV endpoint. *LVEF* left ventricular ejection fraction, *CoV*_*LS*_ longitudinal strain coefficient of variation, *GLS* global longitudinal strain, *CoV*_*CS*_ circumferential strain coefficient of variation, *GCS* global circumferential strain, *CoV*_*RS*_ radial strain coefficient of variation, *GRS* global radial strain, *KM* Kaplan-Meier, *CV* cardiovascular.

**Fig. 6:**
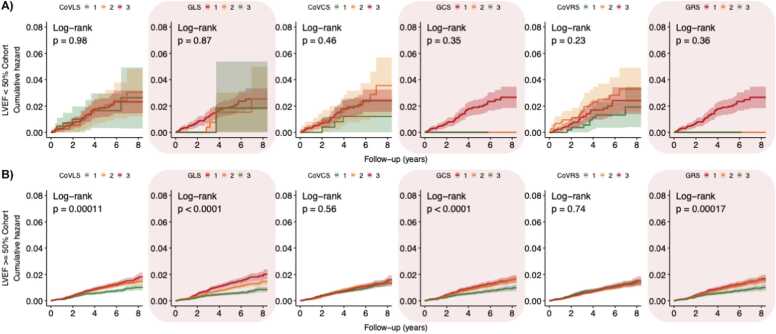
**Strain biomarker KM hazard curves by tertiles for incident MI.** In participants with reduced LVEF < 50% **(panel A),** strain CoV and global strain were not predictive of incident MI. Conversely, in participants with preserved LVEF ≥ 50% **(Panel B),** lower tertiles of GLS*,* GCS*,* GRS and greater tertiles of CoV_LS_ were associated with greater risk of MI. *LVEF* left ventricular ejection fraction, *CoV*_*LS*_ longitudinal strain coefficient of variation, *GLS* global longitudinal strain, *CoV*_*CS*_ circumferential strain coefficient of variation, *GCS* global circumferential strain, *CoV*_*RS*_ radial strain coefficient of variation, *GRS* global radial strain, *KM* Kaplan-Meier, *MI* myocardial infarction.

**Fig. 7:**
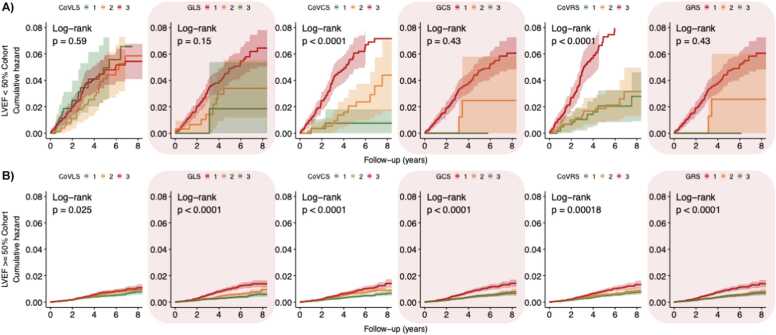
**Strain biomarker KM hazard curves by tertiles for incident HF.** In participants with reduced LVEF < 50% **(panel A),***CoV*_*CS*_ and *CoV*_*RS*_ stratified the risk of incident HF better than global strain. Conversely, in participants with preserved LVEF ≥ 50% **(Panel B),** the greatest tertile of CoV_CS_ and CoV_RS_ had comparable HF event numbers to the lowest tertile of GLS*,* GCS and GRS. *LVEF* left ventricular ejection fraction, *CoV*_*LS*_ longitudinal strain coefficient of variation, *GLS* global longitudinal strain, *CoV*_*CS*_ circumferential strain coefficient of variation, *GCS* global circumferential strain, *CoV*_*RS*_ radial strain coefficient of variation, *GRS* global radial strain, *KM* Kaplan-Meier, *HF* heart failure

**Fig. 8:**
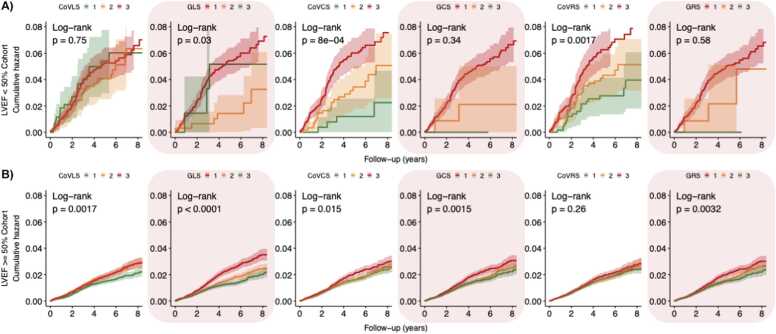
**Strain biomarker KM hazard curves by tertiles for incident arrhythmia.** In participants with reduced LVEF < 50% **(panel A),** CoV_CS_ and CoV_RS_ stratified the risk of incident arrhythmia better than global strain. Conversely, in participants with preserved LVEF ≥ 50% **(Panel B),** GLS*,* GCS and GRS stratified the risk of incident arrhythmia better than their strain CoV counterparts. *LVEF* left ventricular ejection fraction, *CoV*_*LS*_ longitudinal strain coefficient of variation, *GLS* global longitudinal strain, *CoV*_*CS*_ circumferential strain coefficient of variation, *GCS* global circumferential strain, *CoV*_*RS*_ radial strain coefficient of variation, *GRS* global radial strain, *KM* Kaplan-Meier

**Fig. 9:**
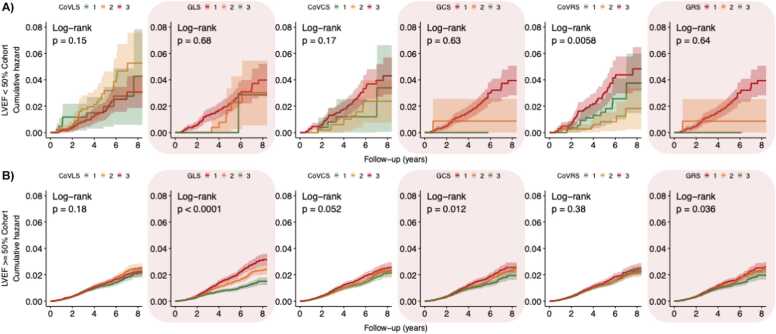
**Strain biomarker KM hazard curves by tertiles for death from any cause.** In participants with reduced LVEF < 50% **(panel A),** CoV_RS_ stratified the risk of death from any cause better than other strain markers. Conversely, in participants with preserved LVEF ≥ 50% **(Panel B),***GLS* was the most sensitive marker for increased risk of all-cause death. *LVEF* left ventricular ejection fraction, *CoV*_*LS*_ longitudinal strain coefficient of variation, *GLS* global longitudinal strain, *CoV*_*CS*_ circumferential strain coefficient of variation, *GCS* global circumferential strain, *CoV*_*RS*_ radial strain coefficient of variation, *GRS* global radial strain, *KM* Kaplan-Meier

Fig. 5, Fig. 6, Fig. 7, Fig. 8, Fig. 9 present the unadjusted Kaplan-Meier cumulative hazards by strain biomarker tertiles, stratified by LVEF for the composite CV endpoint, MI, HF, arrhythmia and death from any cause. In participants with preserved LVEF ≥ 50%, the lowest tertiles of GLS, GCS and GRS were associated with the greatest risk of developing the listed endpoints (P < 0.05 for all, respectively). The clear separation between GLS tertile curves suggested that GLS was the strongest predictor. By comparison, in participants with reduced LVEF < 50%, CoV_CS_ and CoV_RS_ revealed the greatest differences across three tertiles with clear separation of hazard curves for risk of HF, arrhythmia and the composite CV endpoint (P < 0.05 for all, respectively). CoV_RS_ had the strongest association with death from any cause; participants in the lowest and highest tertiles did worse than participants in the middle tertile (P = 0.0058).

## 4 DISCUSSION

To our knowledge, this is the first study to investigate the prognostic role of novel strain heterogeneity measurements derived from machine learning-based CMR image analysis in a prospectively enrolled general population. Our major finding is that CoV_CS_ and CoV_RS_ provide independent and incremental risk predictions for all-cause death and a combined CV endpoint, including HF and arrhythmia in multivariable regression models adjusted for established clinical and CMR markers of CV risk, including global strain and known regional strain abnormalities **(Central Illustration).** Notably, greater CoV_CS_ and CoV_RS_ strongly prognosticated incident HF in participants with 1) reduced LVEF < 50% and 2) preserved LVEF ≥ 50% in addition to at least 3 of > 65 years of age, obesity, hypertension, diabetes, CKD and AF, underscoring the significance of functional heterogeneity in cardiac disease.

Although disease affects cardiac function heterogeneously, imaging-based assessment of LV function is heavily focused on global quantification (e.g. LVEF and GLS*)*. Qualitative descriptions of regional abnormalities also have high intra-, inter-observer variability and low test-retest reproducibility. [41] There is an unmet need for a precise, reliable and reproducible quantitative measure of contractile regionality severity. In a cohort of greater than 55,000 predominantly Caucasian participants, we demonstrated that the CMR-FT derived strain heterogeneity markers CoV_CS_ and CoV_RS_ were strongly predictive of hospitalization for HF, moderately predictive of arrhythmia events and weakly predictive of death from any cause after adjusting for age, sex, conventional CV risk factors (e.g. diabetes and hypertension), clinically-integrated CMR biomarkers (e.g. LVMi, LVEDVi and LVEF), global strain and abnormal regional strain.

Our findings are consistent with the published, albeit limited, literature on strain heterogeneity. In several small case-control studies of ischemic, non-ischemic cardiomyopathies, hypertrophic cardiomyopathy (HCM) and channelopathies, Haugaa, Haland and colleagues have shown that mechanical dispersion, a marker of cardiac conduction heterogeneity was superior to LVEF and GLS in risk assessment of ventricular arrhythmias. [12], [13], [14], [15], [17] The novelty of our study is derived from the assessment of a marker that measures heterogeneity of myocardial mechanical deformation, not electrical activation **(**Fig. 1**).** Dilated cardiomyopathy with left bundle branch block may have greater mechanical dispersion despite low strain CoV; conversely, an anterior MI may present with greater strain CoV despite normal range mechanical dispersion. A previous case-control study of 22 patients with HCM and preserved LVEF demonstrated the importance of strain CoV; CoV_CS*,*_ was superior to *GCS* in identifying extensive fibrosis and therefore higher risk of sudden cardiac death in HCM. [18] Future studies should investigate the relationship between strain CoV and mechanical dispersion, two complementary strain heterogeneity biomarkers.

Importantly, we build on the work by others, including colleagues Chadalavada *et al.* in the field of deformation imaging. [6] In our study, GLS stands out as the strongest predictor of future CV events amongst healthy subgroups, defined by ICD diagnoses and LVEF respectively. Conversely, CoV_CS_ and CoV_RS_ provided noticeably greater risk stratification of participants with reduced LVEF < 50% for incident HF and arrhythmia; and were more predictive of HF amongst participants with preserved LVEF ≥ 50% and ≥ 3 risk factors for HFpEF. These findings as well as the weaker or absent association between CoV_LS_, GCS*,* GRS and study endpoints excluding HF, could be explained by their relationship to myocardial fiber arrangement and contraction.

First, circumferential and radial contraction is more relevant to LV function than longitudinal function as the ratio of circumferentially to longitudinally orientated fibers increases towards the base. [5], [42] Second, circumferential and radial shortening are the result of complex interactions between myocardial fibers in the mid to subepicardial layers with significant epi- to endocardial and basal to apical gradients whereas longitudinal shortening by the subendocardium, the most vulnerable myocardium is relatively homogenous across the wall **(Central Illustration).** [5], [42], [43] It is, therefore, highly plausible that only GLS, CoV_CS_ and CoV_RS_ measure the pertinent cardiac physiology across the respective strain vector as markers of global and heterogeneous LV function, respectively, rendering them highly sensitive and specific markers of cardiac (dys)function.

There are further noteworthy observations. Although ischemic cardiomyopathy is the archetype heterogeneous cardiac disease, the absence of an association between CoV and incident MI should perhaps be expected. [5], [44] Myocardial dysfunction and changes in strain would develop at the time of coronary occlusion, ischemia and infarction (ischemic cascade), whereas cardiac remodeling, atrial and ventricular, can precede the development of arrhythmia and HF. [3], [44] We must also treat the non-significant association of lower CoV_LS_ with death from any cause and the composite CV endpoint, including arrhythmia and HF, cautiously. This is because CoV_LS_ was directly proportional to risk on univariable analysis and only inversely proportional to risk after adjusting for CMR markers, including GLS. On the other hand, low CoV_LS_ might indirectly measure severely impaired longitudinal function in cardiac disease.

## 5 LIMITATIONS

In addition to inherent biases of an observational study, including confounding and reverse causation, the generalizability of our findings is limited by a predominantly White, healthier and above average physically active population at recruitment. We were underpowered to compare the prognostic importance of strain CoV in prevalent focal versus diffuse disease. Nevertheless, the expected low event rates for MI, HF, arrhythmia and death from any cause render our statistically significant associations highly relevant. In enriched populations, the prognostic power may be higher.

In comparison to global strain, strain CoV calculation is more prone to propagation error when dividing the SD by the mean of 16 sometimes incomplete machine learning-derived segmental strain values. A recent review by Smiseth *et al.* raised the valid concern that segmental strain is more susceptible to artifact. [11] Our use of a statistical outlier methodology that was validated against stringent visual assessment of greater than 1000 CMR-FT scans with >97% considered at least acceptable instills confidence in our results. [31] We also posit that machine learning-driven advancements in automated image analysis will improve the precision and reliability of segmental strain and strain CoV. Furthermore, we acknowledge that CMR-FT and its ultrasound comparator, speckle tracking echocardiography, have their advantages and disadvantages. Speckle tracking echocardiography may theoretically quantify regional strain more accurately by tracking ultrasonic interference patterns or “speckles” across the thickness of the myocardium in comparison to feature tracking along predominantly thin endocardial contours in CMR-FT. [1], [5], [28] We contend that these weaknesses are more than offset by the superior signal-to-noise ratio, plane reproducibility and myocardial coverage of CMR. [1], [5], [31].

Finally, we modeled a composite CV endpoint rather than the archetype major adverse cardiovascular event (MACE), which typically includes stroke. [45] Our central aim to develop cardiac-specific heterogeneity biomarkers justifies this decision, a priori.

Increased appreciation of especially early cardiac disease regionality can advance our understanding of cardiac disease pathogenesis and hopefully, translate into more tailored CV health care prevention in the era of personalized medicine. As one of the first quantitative cardiac heterogeneity biomarkers, CMR machine learning-derived strain CoV in the circumferential and radial directions is prognostic of incident CV events in a general population. We propose future validation of strain CoV 1) in other population studies to establish stratified normal reference ranges; 2) in diseased cohorts to assess utility in established clinical pathways and to assess test-retest reproducibility when image acquisition is suboptimal; and 3) by using the more accessible and cost-effective speckle tracking echocardiography. The exponential expansion and integration of machine learning-assisted medical image analysis makes strain CoV imminently translatable into routine clinical practice.

## 6 CONCLUSIONS

Strain heterogeneity biomarkers have a role in CV event risk stratification in the future. We highlighted that strain CoV in the circumferential (CoV_CS._) and radial (CoV_RS_) directions predicts HF, arrhythmia and death from any cause independent of CV risk factors, known regional cardiac dysfunction, established CMR biomarkers and global strain in a general population*.* The proliferation of machine learning-based CMR image analysis provides opportunities for strain CoV to have a role in future cardiology guidelines and practice. Results need to be validated in other community-based populations, tested in diseased cohorts and assessed using more accessible, low-cost speckle tracking echocardiography.

## Sources of funding

K.H. was supported by a British Heart Foundation Clinical Research Training Fellowship no. FS/CRTF/23/24428. S.C. and S.E.P. have received funding from the European Union’s Horizon 2020 research and innovation program under grant agreement No. 825903 (euCanSHare project). K.F. was supported by the Medical College of Saint Bartholomew’s Hospital Trust, an independent registered charity that promotes and advances medical and dental education and research at Barts and The London School of Medicine and Dentistry. S.E.P. acknowledges the British Heart Foundation for funding the manual analysis to create a cardiovascular magnetic resonance imaging reference standard for the UK Biobank imaging resource in 5000 CMR scans (www.bhf.org.uk; PG/14/89/31194). S.E.P. acknowledges support from the “SmartHeart” EPSRC program grant (www.nihr.ac.uk; EP/P001009/1). N.A. acknowledges support from Medical Research Council for his Clinician Scientist Fellowship (MR/X020924/1). This work acknowledges the support of the National Institute for Health and Care Research Barts Biomedical Research Centre (NIHR203330); a delivery partnership of Barts Health NHS Trust, Queen Mary University of London, St George’s University Hospitals NHS Foundation Trust and St George’s University of London. Barts Charity (G-002346) contributed to fees required to access UK Biobank data [access application #2964]. This article is supported by the London Medical Imaging and Artificial Intelligence Centre for Value Based Healthcare (AI4VBH), which is funded from the Data to Early Diagnosis and Precision Medicine strand of the government’s Industrial Strategy Challenge Fund, managed and delivered by Innovate UK on behalf of UK Research and Innovation (UKRI). Views expressed are those of the authors and not necessarily those of the AI4VBH Consortium members, the NHS, Innovate UK, or UKRI. The funders provided support in the form of salaries for authors as detailed above, but did not have any additional role in the study design, data collection and analysis, decision to publish, or preparation of the manuscript.

## CRediT authorship contribution statement

**Kerrick Hesse:** Writing – original draft, Visualization, Methodology, Investigation, Formal analysis, Data curation, Conceptualization. **Mohammed Y Khanji:** Writing – review and editing, Supervision. **C. Anwar A Chahal:** Writing – review and editing, Supervision. **Jose D. Vargas:** Supervision. **Jose Paiva:** Data curation. **Sucharitha Chadalavada:** Data curation. **Kenneth Fung:** Data curation. **Steffen E. Petersen:** Writing – review and editing, Supervision, Conceptualization. **Nay Aung:** Writing – review and editing, Supervision, Conceptualization.

## Declaration of Competing Interest

The authors declare the following financial interests/personal relationships which may be considered as potential competing interests: Kerrick Hesse reports financial support was provided by British Heart Foundation. Steffen Petersen reports a relationship with Circle that includes: consulting or advisory. If there are other authors, they declare that they have no known competing financial interests or personal relationships that could have appeared to influence the work reported in this paper.

## Data Availability

This research was conducted using the UK Biobank resource under access application 2964. UK Biobank will make the data available to all *bona fide* researchers for all types of health-related research that is in the public interest, without preferential or exclusive access for any persons. All researchers will be subject to the same application process and approval criteria as specified by UK Biobank. For more details on the access procedure, see the UK Biobank website: http://www.ukbiobank.ac.uk/register-apply/.

## Footnotes

Appendix A Supplemental data associated with this article can be found in the online version at doi:10.1016/j.jocmr.2025.101976.
