# Supplementary material for: Novel cardiovascular magnetic resonance strain heterogeneity phenotypes predict cardiovascular events: A prospective UK Biobank study
Source: J Cardiovasc Magn Reson. 2025 Oct 29;27(2):101976. doi: 10.1016/j.jocmr.2025.101976 (PMC12766616; doi:10.1016/j.jocmr.2025.101976)
Supplement: Supplementary file 1 — Supplemental material [file mmc1.docx]

**SUPPLEMENTAL APPENDIX**

**Table of Contents**

[Supplemental Table 1. Important disease phenotypes, cross-referenced with the structure and content of available UKBB data. 2](#_Toc207453750)

[Supplemental Table 2. Variance inflation factor of predictor variables in regression models 3 by endpoint and strain categories. 17](#_Toc207453751)

[Supplemental Table 3. Results of Cox proportional hazards regression models to predict a composite CV endpoint by global & CoV strain. 19](#_Toc207453752)

[Supplemental Table 4. Results of Cox proportional hazards regression models to predict incident MI by global & CoV strain. 20](#_Toc207453753)

[Supplemental Table 5. Results of Cox proportional hazards regression models to predict incident HF by global & CoV strain. 21](#_Toc207453754)

[Supplemental Table 6. Results of Cox proportional hazards regression models to predict incident arrhythmia by global & CoV strain. 22](#_Toc207453755)

[Supplemental Table 7. Results of Cox proportional hazards regression models to predict death from any cause by global & CoV strain. 23](#_Toc207453756)

[Supplemental Figure 1. Strain CoV distribution, stratified by number of valid LV segments. 25](#_Toc207453757)

[Supplemental Figure 2. CMR biomarker A) density and B) QQ plots. 26](#_Toc207453758)

[Supplemental Figure 3. Incident composite CV endpoint residual *vs.* time plots. 27](#_Toc207453759)

[Supplemental Figure 4. Incident myocardial infarction residual *vs.* time plots. 29](#_Toc207453760)

[Supplemental Figure 5. Incident heart failure residual *vs.* time plots. 31](#_Toc207453761)

[Supplemental Figure 6. Incident arrhythmia residual *vs.* time plots. 33](#_Toc207453762)

[Supplemental Figure 7. Death from any cause residual *vs.* time plots. 35](#_Toc207453763)

[Supplemental Figure 8. Study flowchart, summarizing UKBB participant inclusion criteria. 37](#_Toc207453764)

[Supplemental Figure 9. Correlogram of CMR biomarkers, including strain CoV. 38](#_Toc207453765)

**Tables**

# Supplemental Table 1. Important disease phenotypes, cross-referenced with the structure and content of available UKBB data.

| **Phenotype** | **Data fields** | **Field names** | **Data codes** | **Data code definitions** |
| --- | --- | --- | --- | --- |
| Aortic stenosis | 20002 | Non-cancer illness code, self-reported | 1490 | Aortic stenosis |
|  | 41202  41204  41270  40001  40002 | Diagnoses – main ICD10  Diagnoses – secondary ICD10  Diagnoses – ICD10  Underlying (primary) cause of death: ICD10  Contributory (secondary) cause of death: ICD10 | I06.0, I06.2, I35.0, I35.2 | Rheumatic aortic stenosis, Rheumatic aortic stenosis with insufficiency, Aortic (valve) stenosis, Aortic (valve) stenosis with insufficiency |
|  | 41200  41210  41272 | Operative procedures – main OPCS4  Operative procedures – secondary OPCS4  Operative procedures - OPCS4 | K35.2 | Percutaneous transluminal aortic valvotomy |
|  | 41256  41258  41273 | Operative procedures – main OPCS3  Operative procedures – secondary OPCS3  Operative procedures – OPCS3 | 314.2 | Other valvuloplasty: aortic valve |
| Arrhythmogenic cardiomyopathy | 41202  41204  41270  40001  40002 | Diagnoses – main ICD10  Diagnoses – secondary ICD10  Diagnoses – ICD10  Underlying (primary) cause of death: ICD10  Contributory (secondary) cause of death: ICD10 | I42.8 | Other cardiomyopathies |
| Atrial fibrillation  or flutter | 20002 | Non-cancer illness code, self-reported | 1471, 1483 | Atrial fibrillation, Atrial flutter |
|  | 41202  41204  41270  40001  40002 | Diagnoses – main ICD10  Diagnoses – secondary ICD10  Diagnoses – ICD10  Underlying (primary) cause of death: ICD10  Contributory (secondary) cause of death: ICD10 | I48, I48.0, I48.1, I48.2, I48.3, I48.4, I48.9 | Atrial fibrillation and flutter, Paroxysmal atrial fibrillation, Persistent atrial fibrillation, Chronic atrial fibrillation, Typical atrial flutter, Atypical atrial flutter, Atrial fibrillation and atrial flutter, unspecified |
|  | 41203  41205  41271 | Diagnoses – main ICD9  Diagnoses – secondary ICD9  Diagnoses – ICD9 | 4273 | Atrial fibrillation and flutter |
|  | 41200  41210  41272 | Operative procedures – main OPCS4  Operative procedures – secondary OPCS4  Operative procedures - OPCS4 | K57.1, K62.1, K62.2, K62.3, K62.4 | Percutaneous transluminal ablation of atrioventricular node, Percutaneous transluminal ablation of pulmonary vein to left atrium conducting system, Percutaneous transluminal ablation of atrial wall for atrial flutter, Percutaneous transluminal ablation of conducting system of heart for atrial flutter NEC, Percutaneous transluminal internal cardioversion NEC |
| Bradyarrhythmia | 20002 | Non-cancer illness code, self-reported | 1486 | Sick sinus syndrome |
|  | 20004 | Operation code, self-reported | 1548 | Pacemaker insertion |
|  | 41202  41204  41270  40001  40002 | Diagnoses – main ICD10  Diagnoses – secondary ICD10  Diagnoses – ICD10  Underlying (primary) cause of death: ICD10  Contributory (secondary) cause of death: ICD10 | I44, I44.1, I44.2, I44.3, I44.5, I49.5 | Atrioventricular and left bundle-branch block, Atrioventricular block, second degree, Atrioventricular block, complete, Other and unspecified atrioventricular block, Other specified heart block, Sick sinus syndrome |
|  | 41203  41205  41271 | Diagnoses – main ICD9  Diagnoses – secondary ICD9  Diagnoses – ICD9 | 4260, 4261, 4266 | Atrioventricular block, complete, Atrioventricular block, other and unspecified, Other specified heart block |
|  | 41200  41210  41272 | Operative procedures – main OPCS4  Operative procedures – secondary OPCS4  Operative procedures - OPCS4 | K60, K60.1, K60.2, K60.3, K60.4, K60.5, K60.6, K60.8, K60.9, K61, K61.1, K61.2,  K61.3, K61.4, K61.5, K61.6, K61.8, K61.9  K73, K73.1, K73.2, K73.3, K73.8, K73.9, K74, K74.1, K74.2, K74.8, K74.9 | Cardiac pacemaker system introduced through vein, Implantation of intravenous cardiac pacemaker system NEC, Resiting of lead of intravenous cardiac pacemaker system, Renewal of intravenous cardiac pacemaker system, Removal of intravenous cardiac pacemaker system, Implantation of intravenous single chamber cardiac pacemaker system, Implantation of intravenous dual chamber cardiac pacemaker system, Other specified cardiac pacemaker system introduced through vein, Unspecified cardiac pacemaker system introduced through vein, Other cardiac pacemaker system, Implantation of cardiac pacemaker system NEC, Resiting of lead of cardiac pacemaker system NEC, Renewal of cardiac pacemaker system NEC, Removal of cardiac pacemaker system NEC, Implantation of single chamber cardiac pacemaker system, Implantation of dual chamber cardiac pacemaker system, Other specified other cardiac pacemaker system, Unspecified other cardiac pacemaker system,  Other cardiac pacemaker system introduced through vein, Renewal of intravenous single chamber cardiac pacemaker system, Renewal of intravenous dual chamber cardiac pacemaker system, Other specified other cardiac pacemaker system introduced through vein, Unspecified other cardiac pacemaker system introduced through vein, Cardiac pacemaker system, Renewal of single chamber cardiac pacemaker system NEC, Renewal of dual chamber cardiac pacemaker system NEC, Other specified cardiac pacemaker system, Unspecified cardiac pacemaker system |
|  | 41256  41258  41273 | Operative procedures – main OPCS3  Operative procedures – secondary OPCS3  Operative procedures – OPCS3 | 305 | Insertion of pace-maker |
| Cardiomyopathy | 20002 | Non-cancer illness code, self-reported | 1079 | cardiomyopathy |
|  | 41202  41204  41270  40001  40002 | Diagnoses – main ICD10  Diagnoses – secondary ICD10  Diagnoses – ICD10  Underlying (primary) cause of death: ICD10  Contributory (secondary) cause of death: ICD10 | I42.0, I42.1, I42.2, I42.3, I42.3, I42.4, I42.5, I42.6, I42.7, I42.8, I42.9, I43.0, I43.1, I43.2, I43.8 | Dilated cardiomyopathy, Obstructive hypertrophic cardiomyopathy, Other hypertrophic cardiomyopathy, Endomyocardial (eosinophilic) disease, Endocardial fibroelastosis, Other restrictive cardiomyopathy, Alcoholic cardiomyopathy, Cardiomyopathy due to drugs and other external agents, Other cardiomyopathies, Cardiomyopathy, unspecified, Cardiomyopathy in infectious and parasitic diseases classified elsewhere, Cardiomyopathy in metabolic disease, Cardiomyopathy in nutritional diseases, Cardiomyopathy in other diseases classified elsewhere |
|  | 41203  41205  41271 | Diagnoses – main ICD9  Diagnoses – secondary ICD9  Diagnoses – ICD9 | 4251, 4254 | Hypertrophic obstructive cardiomyopathy, Other primary cardiomyopathies |
|  | 41200  41210  41272 | Operative procedures – main OPCS4  Operative procedures – secondary OPCS4  Operative procedures - OPCS4 | K16.6, K24.5, K24.6, K24.7 | Percutaneous transluminal chemical mediated septal ablation, Relief of left ventricular outflow tract obstruction, Myectomy of LVOT, Myotomy of LVOT |
| Cerebrovascular disease/Stroke | 20002 | Non-cancer illness code, self-reported | 1081, 1082, 1491, 1986 | Stroke, Transient ischaemic attack (tia), Brain haemorrhage, Subarachnoid haemorrhage, |
|  | 20004 | Operation code, self-reported | 1105, 1106, 1109 | Carotid artery surgery/endarterectomy, Cerebral artery aneurysm surgery or clipping, Carotid artery angioplasty +/- stent |
|  | 41202  41204  41270  40001  40002 | Diagnoses – main ICD10  Diagnoses – secondary ICD10  Diagnoses – ICD10  Underlying (primary) cause of death: ICD10  Contributory (secondary) cause of death: ICD10 | G46, I60, I61, I62, I63, I64 | Vascular syndromes of brain in cerebrovascular diseases, Subarachnoid haemorrhage, Intracerebral haemorrhage, Other nontraumatic intracranial haemorrhage, Cerebral infarction, Stroke not specified as haemorrhage or infarction |
|  | 41203  41205  41271 | Diagnoses – main ICD9  Diagnoses – secondary ICD9  Diagnoses – ICD9 | 430, 431, 433, 434, 436 | Subarachnoid haemorrhage, Intracerebral haemorrhage, Occlusion and stenosis of precerebral arteries, Occlusion of cerebral arteries, Acute but ill-defined cerebrovascular disease |
|  | 41200  41210  41272 | Operative procedures – main OPCS4  Operative procedures – secondary OPCS4  Operative procedures - OPCS4 | V03.7 | Decompressive craniectomy |
| Chronic kidney disease | 20002 | Non-cancer illness code, self-reported | 1192, 1193, 1194, 1196, 1197, 1200, 1405, 1427, 1519, 1520, 1607, 1608, 1609 | Renal/kidney failure, Renal failure requiring dialysis, Renal failure not requiring dialysis, Urinary tract infection/kidney infection, Kidney stone/ureter stone/bladder stone, Ureteric obstruction/hydronephrosis, Other renal/kidney problem, Polycystic kidney, Kidney nephropathy, Iga nephropathy, Diabetic nephropathy, Nephritis, Glomerulonephritis, |
|  | 20004 | Operation code, self-reported | 1195, 1197, 1487, 1580, 1581, 1582, 1618 | Renal/kidney transplant, Percutaneous/open kidney stone surgery/lithotripsy, Nephrectomy/kidney removed, Dialysis access surgery, Haemodialysis access / fistula surgery, Peritoneal dialysis (capd) access surgery, Renal biopsy/kidney biopsy |
|  | 41202  41204  41270  40001  40002 | Diagnoses – main ICD10  Diagnoses – secondary ICD10  Diagnoses – ICD10  Underlying (primary) cause of death: ICD10  Contributory (secondary) cause of death: ICD10 | I12, I13, N00,  N01, N02, N03, N04, N05, N06, N07, N08, N10, N11, N12, N13, N14, N15, N16, N17. N18, N19, N20, N21, N22  N23, N25, N26, N27, N28, N29, N31, C64, C65 | Hypertensive renal disease, Hypertensive heart and renal disease, Acute nephritic syndrome, Rapidly progressive nephritic syndrome, Recurrent and persistent haematuria, Chronic nephritic syndrome, Nephrotic syndrome, Unspecified nephritic syndrome, Isolated proteinuria with specified morphological lesion, Hereditary nephropathy, not elsewhere specified, Glomerular disorders in diseases classified elsewhere, Acute tubulo-interstitial nephritis, Chronic tubule-interstitial nephritis, Tubulo-interstitial nephritis, not specified as acute or chronic, Obstructive and reflux uropathy, Drug- and heavy-metal-induced tubule-interstitial and tubular conditions, Other renal tubule-interstitial diseases, Renal tubule-interstitial disorders in disease classified elsewhere, Acute renal failure, Chronic renal failure, Unspecified renal failure, Calculus of kidney and ureter, Calculus of lower urinary tract, Calculus of urinary tract in diseases classified elsewhere, Unspecified renal colic, Disorders resulting from impaired renal tubular function, Unspecified contracted kidney, Small kidney of unknown cause, Other disorders of kidney and ureter, not elsewhere classified, Other disorders of kidney and ureter in diseases classified elsewhere, Neuromuscular dysfunction of bladder, not elsewhere classified, Malignant neoplasm of kidney, except renal pelvis, Malignant neoplasm of renal pelvis |
|  | 41203  41205  41271 | Diagnoses – main ICD9  Diagnoses – secondary ICD9  Diagnoses – ICD9 | 189, 403, 581, 582, 583, 584, 585, 586, 587, 588, 589, 590, 591, 592, 593 | Malignant neoplasm of kidney and other and unspecified urinary organs, Hypertensive renal disease, Nephrotic syndrome, Chronic glomerulonephritis, Nephritis and nephropathy, not specified as acute or chronic, Acute renal failure, Chronic renal failure, Renal failure, unspecified, Renal sclerosis, unspecified, Disorders resulting from impaired renal function, Small kidney of unknown cause, Infections of kidney, Hydronephrosis, Calculus of kidney and ureter, Other disorders of kidney and ureter |
|  | 41200  41210  41272 | Operative procedures – main OPCS4  Operative procedures – secondary OPCS4  Operative procedures - OPCS4 | M01, M02, M03, M04, M05, M06, M08, M09, M10, M11, M13, M14, M15, M16, M17 | Transplantation of kidney, Total excision of kidney, Partial excision of kidney, Open extirpation of lesion of kidney, Open repair of kidney, Incision of kidney, Other open operations on kidney, Therapeutic endoscopic operations on calculus of kidney, Other therapeutic endoscopic operations on kidney, Diagnostic endoscopic examination of kidney, Percutaneous puncture of kidney, Extracorporeal fragmentation of calculus of kidney, Operations on kidney along nephrostomy tube track, Other operations on kidney, Interventions associated with transplantation of kidney |
|  | 41256  41258  41273 | Operative procedures – main OPCS3  Operative procedures – secondary OPCS3  Operative procedures – OPCS3 | 560, 561, 562, 563, 564, 565, 566, 567, 568, 569, 571 | Nephrotomy, not elsewhere classified : biopsy, not elsewhere classified, Nephrostomy and pyelostomy, Pyelostomy, Removal of renal calculus, Percutaneous puncture of kidney, Removal of kidney, complete, Transplantation of kidney, Excision or destruction of lesion of kidney, Repair of kidney and renal pelvis, Nephropexy, Renal arterial and venous puncture |
| Coronary artery disease | 20002 | Non-cancer illness code, self-reported | 1075 | Heart attack/myocardial infarction |
|  | 20004 | Operation code, self-reported | 1070, 1095 | Coronary angioplasty (ptca) +/- stent, Coronary artery bypass grafts (cabg) |
|  | 41202  41204  41270  40001  40002 | Diagnoses – main ICD10  Diagnoses – secondary ICD10  Diagnoses – ICD10  Underlying (primary) cause of death: ICD10  Contributory (secondary) cause of death: ICD10 | I20, I21, I22, I23, I24, I25 | Angina pectoris, Acute myocardial infarction, Subsequent myocardial infarction, Certain current complications following acute myocardial infarction, Other acute ischaemic heart diseases, Chronic ischaemic heart disease |
|  | 41203  41205  41271 | Diagnoses – main ICD9  Diagnoses – secondary ICD9  Diagnoses – ICD9 | 410, 411, 412, 413, 414 | Acute myocardial infarction, Other acute and subacute forms of ischaemic heart disease, Old myocardial infarction, Angina pectoris, Other forms of chronic ischaemic heart disease |
|  | 41200  41210  41272 | Operative procedures – main OPCS4  Operative procedures – secondary OPCS4  Operative procedures - OPCS4 | K40, K41, K42, K43, K44, K45, K46, K47, K49, K50, K51, K75 | Saphenous vein graft replacement of coronary artery, Other autograft replacement of coronary artery, Allograft replacement of coronary artery, Prosthetic replacement of coronary artery, Other replacement of coronary artery, Connection of thoracic artery to coronary artery, Other bypass of coronary artery, Repair of coronary artery, Transluminal balloon angioplasty of coronary artery, Other therapeutic transluminal operations on coronary artery, Diagnostic transluminal operations on coronary artery, Percutaneous transluminal balloon angioplasty and insertion of stent into coronary artery |
|  | 41256  41258  41273 | Operative procedures – main OPCS3  Operative procedures – secondary OPCS3  Operative procedures – OPCS3 | 304.1, 304.2, 304.3 | Operations affecting myocardium : coronary endarterectomy, Operations affecting myocardium : revascularization (poudrage), Operations affecting myocardium : coronary anastomosis or graft |
| Diabetes | 20002 | Non-cancer illness code, self-reported | 1220, 1222, 1223 | Diabetes, Type 1 diabetes, Type 2 diabetes |
|  | 41202  41204  40001  40002 | Diagnoses – main ICD10  Diagnoses – secondary ICD10  Underlying (primary) cause of death: ICD10  Contributory (secondary) cause of death: ICD10 | E10, E11, E12, E13, E14 | Insulin-dependent diabetes mellitus, Non-insulin-dependent diabetes mellitus, Malnutrition-related diabetes mellitus, Other specified diabetes mellitus, Unspecified diabetes mellitus |
|  | 41203  41205  41271 | Diagnoses – main ICD9  Diagnoses – secondary ICD9  Diagnoses – ICD9 | 250 | Diabetes mellitus |
|  | 41200  41210  41272 | Operative procedures – main OPCS4  Operative procedures – secondary OPCS4  Operative procedures - OPCS4 | J54.2, J54.4 | Transplantation of whole pancreas, Transplantation of islet of Langerhans |
|  | 6153 | Medication for cholesterol, blood pressure, diabetes, or take exogenous hormones | 3 | Insulin |
|  | 6177 | Medication for cholesterol, blood pressure or diabetes | 3 | Insulin |
|  | 20003 | Treatment/medication code | 1140874744, 1140884600, 1141171646 | Gliclazide, metformin, pioglitazone |
| Dilated cardiomyopathy | 41202  41204  41270  40001  40002 | Diagnoses – main ICD10  Diagnoses – secondary ICD10  Diagnoses – ICD10  Underlying (primary) cause of death: ICD10  Contributory (secondary) cause of death: ICD10 | I42.0 | Dilated cardiomyopathy |
| Dyslipidemia | 20002 | Non-cancer illness code, self-reported | 1473 | High cholesterol |
|  | 41202  41204  40001  40002 | Diagnoses – main ICD10  Diagnoses – secondary ICD10  Underlying (primary) cause of death: ICD10  Contributory (secondary) cause of death: ICD10 | E78 | Disorders of lipoprotein metabolism and other lipidaemias |
|  | 41203  41205  41271 | Diagnoses – main ICD9  Diagnoses – secondary ICD9  Diagnoses – ICD9 | 2720, 2721, 2722 | Pure hypercholesterolaemia, Hyperglyceridaemia, Mixed hyperlipidaemia |
|  | 6153 | Medication for cholesterol, blood pressure, diabetes, or take exogenous hormones | 1 | Cholesterol lowering medication |
|  | 6177 | Medication for cholesterol, blood pressure or diabetes | 1 | Cholesterol lowering medication |
|  | 20003 | Treatment/medication code | 1141146234, 1141192736,  1140861954,  1140888648,  1141192410, 1140861958 | Atorvastatin, ezetimibe, fenofibrate, pravastatin, rosuvastatin, simvastatin, |
| Heart failure | 20002 | Non-cancer illness code, self-reported | 1076 | Heart failure |
|  | 41202  41204  40001  40002 | Diagnoses – main ICD10  Diagnoses – secondary ICD10  Underlying (primary) cause of death: ICD10  Contributory (secondary) cause of death: ICD10 | I11.0, I13.0, I13.2, I.25.5, I42.0, I42.6, I42.7, I50 | Hypertensive heart disease with (congestive) heart failure, Hypertensive heart and renal disease with (congestive) heart failure, Hypertensive heart and renal disease with both (congestive) heart failure and renal failure, Ischaemic cardiomyopathy, Dilated cardiomyopathy, Alcoholic cardiomyopathy, Cardiomyopathy due to drugs and other external agents, Heart failure |
|  | 41203  41205  41271 | Diagnoses – main ICD9  Diagnoses – secondary ICD9  Diagnoses – ICD9 | 428 | Heart failure |
|  | 41200  41210  41272 | Operative procedures – main OPCS4  Operative procedures – secondary OPCS4  Operative procedures - OPCS4 | K01, K02, K54, K56, K59.6, K59.7, K60.7, K61.7, K73.3, K74.3 | Transplantation of heart and lung, Other transplantation of heart, Open heart assist operations, Transluminal heart assist operations, Implantation of cardioverter defibrillator using three electrode leads, Renewal of cardioverter defibrillator using three electrode leads,  Implantation of intravenous biventricular cardiac pacemaker system, Implantation of biventricular cardiac pacemaker system, Renewal of intravenous biventricular cardiac pacemaker, Renewal of biventricular cardiac pacemaker NEC |
|  | 41256  41258  41273 | Operative procedures – main OPCS3  Operative procedures – secondary OPCS3  Operative procedures – OPCS3 | 319.2 | Other operations on open heart : heart assist system |
| Hypertension | 20002 | Non-cancer illness code, self-reported | 1065, 1072 | Essential hypertension, hypertension |
|  | 41202  41204  40001  40002 | Diagnoses – main ICD10  Diagnoses – secondary ICD10  Underlying (primary) cause of death: ICD10  Contributory (secondary) cause of death: ICD10 | I10, I11, I12, I13, I14, I15 | Essential (primary) hypertension, Hypertensive heart disease, Hypertensive renal disease, Hypertensive heart and renal disease, Secondary hypertension |
|  | 41203  41205  41271 | Diagnoses – main ICD9  Diagnoses – secondary ICD9  Diagnoses – ICD9 | 401, 403 | Essential hypertension, Hypertensive renal disease |
|  | 6153 | Medication for cholesterol, blood pressure, diabetes, or take exogenous hormones | 2 | Blood pressure medication |
|  | 6177 | Medication for cholesterol, blood pressure or diabetes | 2 | Blood pressure medication |
| Hypertrophic cardiomyopathy | 20002 | Non-cancer illness code, self-reported | 1588 | Hypertrophic cardiomyopathy (hcm/hocm) |
|  | 41202  41204  41270  40001  40002 | Diagnoses – main ICD10  Diagnoses – secondary ICD10  Diagnoses – ICD10  Underlying (primary) cause of death: ICD10  Contributory (secondary) cause of death: ICD10 | I42.1, I42.2 | Obstructive hypertrophic cardiomyopathy, Other hypertrophic cardiomyopathy |
|  | 41203  41205  41271 | Diagnoses – main ICD9  Diagnoses – secondary ICD9  Diagnoses – ICD9 | 4251 | Hypertrophic obstructive cardiomyopathy |
|  | 41200  41210  41272 | Operative procedures – main OPCS4  Operative procedures – secondary OPCS4  Operative procedures - OPCS4 | K16.6, K24.5, K24.6, K24.7 | Percutaneous transluminal chemical mediated septal ablation, Relief of left ventricular outflow tract obstruction, Myectomy of LVOT, Myotomy of LVOT |
| Mitral valve disease | 20002 | Non-cancer illness code, self-reported | 1488, 1489 | Mitral valve prolapse, Mitral stenosis |
|  | 20004 | Operation code, self-reported | 1100 | Mitral valve repair/replacement |
|  | 41202  41204  41270  40001  40002 | Diagnoses – main ICD10  Diagnoses – secondary ICD10  Diagnoses – ICD10  Underlying (primary) cause of death: ICD10  Contributory (secondary) cause of death: ICD10 | I05.0, I05.1, I05.2, I05.8, I05.9, I34.0, I34.1, I34.2, I34.8, I34.9 | Mitral stenosis, Rheumatic mitral insufficiency, Mitral stenosis with insufficiency, Other mitral valve diseases, Mitral valve disease, unspecified, Mitral (valve) insufficiency, Mitral (valve) prolapse, Nonrheumatic mitral (valve) stenosis, Other nonrheumatic mitral valve disorders, Nonrheumatic mitral valve disorder, unspecified |
|  | 41203  41205  41271 | Diagnoses – main ICD9  Diagnoses – secondary ICD9  Diagnoses – ICD9 | 3940, 3942, 3949 | Mitral stenosis, Mitral stenosis with insufficiency, Other and unspecified diseases of mitral valve |
|  | 41200  41210  41272 | Operative procedures – main OPCS4  Operative procedures – secondary OPCS4  Operative procedures - OPCS4 | K25.1, K25.2, K25.3, K25.4, K25.5, K25.8, K25.9, K31.1, K32.1, K30.1 | Allograft replacement of mitral valve, Xenograft replacement of mitral valve, Prosthetic replacement of mitral valve, Replacement of mitral valve NEC23, Mitral valve repair NEC728, Other specified plastic repair of mitral valve, Unspecified plastic repair of mitral valve, Open mitral valvotomy, Closed mitral valvotomy, Revision of plastic repair of mitral valve |
|  | 41256  41258  41273 | Operative procedures – main OPCS3  Operative procedures – secondary OPCS3  Operative procedures – OPCS3 | 313.1, 314.1 | Heart valve replacement : mitral valve , Other valvuloplasty : mitral valve |
| Myocardial infarction | 20002 | Non-cancer illness code, self-reported | 1075 | Heart attack/myocardial infarction |
|  | 41202  41204  41270  40001  40002 | Diagnoses – main ICD10  Diagnoses – secondary ICD10  Diagnoses – ICD10  Underlying (primary) cause of death: ICD10  Contributory (secondary) cause of death: ICD10 | I21, I22, I23, I24.8, I24.9 | Acute myocardial infarction, Subsequent myocardial infarction, Certain current complications following acute myocardial infarction, Other forms of acute ischaemic heart disease, Acute ischaemic heart disease, unspecified |
|  | 41203  41205  41271 | Diagnoses – main ICD9  Diagnoses – secondary ICD9  Diagnoses – ICD9 | 410, 411, 412, 413, 414 | Acute myocardial infarction, Other acute and subacute forms of ischaemic heart disease |
| Myocarditis | 20002 | Non-cancer illness code, self-reported | 1426 | Myocarditis |
|  | 41202  41204  41270  40001  40002 | Diagnoses – main ICD10  Diagnoses – secondary ICD10  Diagnoses – ICD10  Underlying (primary) cause of death: ICD10  Contributory (secondary) cause of death: ICD10 | B33.2, I40.0, I40.1, I40.8, I40.9, I41.0, I41.1, I41.2, I41.8, I51.4, | Viral carditis, Infective myocarditis, Isolated myocarditis, Other acute myocarditis, Acute myocarditis, unspecified, Myocarditis in bacterial diseases classified elsewhere, Myocarditis in viral diseases classified elsewhere, Myocarditis in other infectious and parasitic diseases classified elsewhere, Myocarditis in other diseases classified elsewhere, Myocarditis unspecified |
|  | 41203  41205  41271 | Diagnoses – main ICD9  Diagnoses – secondary ICD9  Diagnoses – ICD9 | 4229, 4290 | Other and unspecified acute myocarditis, Myocarditis unspecified |
| Narrow complex tachycardia | 20002 | Non-cancer illness code, self-reported | 1487 | SVT/ supraventricular tachycardia |
|  | 41202  41204  41270  40001  40002 | Diagnoses – main ICD10  Diagnoses – secondary ICD10  Diagnoses – ICD10  Underlying (primary) cause of death: ICD10  Contributory (secondary) cause of death: ICD10 | I47.1 | Supraventricular tachycardia |
|  | 41203  41205  41271 | Diagnoses – main ICD9  Diagnoses – secondary ICD9  Diagnoses – ICD9 | 4270 | Paroxysmal supraventricular tachycardia |
|  | 41200  41210  41272 | Operative procedures – main OPCS4  Operative procedures – secondary OPCS4  Operative procedures - OPCS4 | K57.2, K57.4, K57.5, K58.1, K58.2, K58.6 | Percutaneous transluminal ablation of conducting system of heart NEC, Percutaneous transluminal ablation of accessory pathway, Percutaneous transluminal ablation of atrial wall NEC, Percutaneous transluminal mapping of conducting system of heart NEC, Percutaneous transluminal electrophysiological studies on conducting system of heart, Percutaneous transluminal three dimensional electroanatomic mapping of conducting system of heart |
| Peripheral  vascular disease | 20002 | Non-cancer illness code, self-reported | 1087 | Leg claudication/ intermittent claudication |
|  | 20004 | Operation code, self-reported | 1102, 1103, 1104, 1108 | Fem-pop bypass/leg artery bypass, Leg artery aneurysm repair, Aortic aneurysm/repair or stent, Leg artery angioplasty +/- stent |
|  | 41202  41204  41270  40001  40002 | Diagnoses – main ICD10  Diagnoses – secondary ICD10  Diagnoses – ICD10  Underlying (primary) cause of death: ICD10  Contributory (secondary) cause of death: ICD10 | I70.0, I70.1, I70.2, I70.8, I70.9, I71.0, I71.1, I71.2, I71.3, I71.4, I71.5, I71.6, I71.8, I71.9 | Atherosclerosis of aorta, Atherosclerosis of renal artery, Atherosclerosis of arteries of the extremities, Atherosclerosis of other arteries, Generalised and unspecified atherosclerosis  Dissection of aorta [any part], Thoracic aortic aneurysm, rupture, Thoracic aortic aneurysm, without mention of rupture, Abdominal aortic aneurysm, ruptured, Abdominal aortic aneurysm, without mention of rupture, Thoracoabdominal aortic aneurysm, ruptured, Thoracoabdominal aortic aneurysm, without mention of rupture, Aortic aneurysm of unspecified site, ruptured, Aortic aneurysm of unspecified site, without mention of rupture |
|  | 41203  41205  41271 | Diagnoses – main ICD9  Diagnoses – secondary ICD9  Diagnoses – ICD9 | 4400, 4401, 4402, 4408, 4409, 4410, 4411, 4414, 4416 | Atherosclerosis of aorta, Atherosclerosis of renal artery, Atherosclerosis of arteries of the extremities, Atherosclerosis of other specified arteries, Atherosclerosis, generalized and unspecified, Dissecting aneurysm (any part), Thoracic aneurysm, ruptured, Abdominal aneurysm without mention of rupture, Aortic aneurysm of unspecified site without mention of rupture |
|  | 41200  41210  41272 | Operative procedures – main OPCS4  Operative procedures – secondary OPCS4  Operative procedures - OPCS4 | L16, L18, L19, L20, L21, L22, L23, L25, L26, L27, L28, L29, L30, L31, L37, L39, L48, L49, L50, L51, L52, L53, L54, L56, L57, L58, L59, L60, L62, L63 | Extra-anatomic bypass of aorta, Emergency replacement of aneurysmal segment of aorta, Other replacement of aneurysmal segment of aorta, Other emergency bypass of segment of aorta, Other bypass of segment of aorta, Attention to prosthesis of aorta, Plastic repair of aorta, Other open operations on aorta, Transluminal operations on aorta, Transluminal insertion of stent graft for aneurysmal segment of aorta, Transluminal operations on aneurysmal segment of aorta, Reconstruction of carotid artery, Other open operations on carotid artery, Transluminal operations on carotid artery, Reconstruction of subclavian artery, Transluminal operations on subclavian artery, Emergency replacement of aneurysmal iliac artery, Other replacement of aneurysmal iliac artery, Other emergency bypass of iliac artery, Other bypass of iliac artery, Reconstruction of iliac artery, Other open operations on iliac artery, Transluminal operations on iliac artery, Emergency replacement of aneurysmal femoral artery, Other replacement of aneurysmal femoral artery, Other emergency bypass of femoral artery, Other bypass of femoral artery, Reconstruction of femoral artery, Other open operations on femoral artery, Transluminal operations on femoral artery |
|  | 41256  41258  41273 | Operative procedures – main OPCS3  Operative procedures – secondary OPCS3  Operative procedures – OPCS3 | 881, 884 | Endarterectomy, Repair of artery |
| Ventricular arrhythmia | 41202  41204  41270  40001  40002 | Diagnoses – main ICD10  Diagnoses – secondary ICD10  Diagnoses – ICD10  Underlying (primary) cause of death: ICD10  Contributory (secondary) cause of death: ICD10 | I46.0, I46.1, I46.9, I47.0, I47.2, I49.0 | Cardiac arrest with successful resuscitation, Sudden cardiac death, so described, Cardiac arrest, unspecified, Reentry ventricular arrhythmia, Ventricular tachycardia, Ventricular fibrillation and flutter |
|  | 41203  41205  41271 | Diagnoses – main ICD9  Diagnoses – secondary ICD9  Diagnoses – ICD9 | 4271, 4274, 4275 | Paroxysmal ventricular tachycardia, Ventricular fibrillation and flutter, Cardiac arrest |
|  | 41200  41210  41272 | Operative procedures – main OPCS4  Operative procedures – secondary OPCS4  Operative procedures - OPCS4 | K57.6, K64.1, X50.3, X50.4, X50.8, X50.9 | Percutaneous transluminal ablation of ventricular wall, Percutaneous radiofrequency ablation of epicardium, External ventricular defibrillation, Other specified external resuscitation, Unspecified external resuscitation |

ICD: International Classification of Diseases; OPCS: Operating Procedure Codes Supplement.

# Supplemental Table 2. Variance inflation factor of predictor variables in regression models 3 by endpoint and strain categories.

| **Strain CoV** | **Global strain** | **Age** | **Sex** | **Ethnicity** | **Smoker** | **BMI** | **Hyperlipidemia** | **HTN** | **Diabetes** | **LVMi** | **LVEDVi** | **LVEF** |
| --- | --- | --- | --- | --- | --- | --- | --- | --- | --- | --- | --- | --- |
| **Composite CV endpoint** | | | | | | | | | | | | |
| **Longitudinal strain** | | | | | | | | | | | | |
| 1.32 | 2.15 | 1.21 | 1.47 | 1.02 | 1.02 | 1.21 | 1.40 | 1.37 | 1.13 | 2.75 | 2.36 | 1.92 |
| **Circumferential strain** | | | | | | | | | | | | |
| 1.56 | 3.55 | 1.19 | 1.48 | 1.02 | 1.01 | 1.21 | 1.40 | 1.37 | 1.13 | 2.59 | 2.17 | 3.07 |
| **Radial strain** | | | | | | | | | | | | |
| 1.12 | 2.92 | 1.18 | 1.48 | 1.02 | 1.01 | 1.21 | 1.40 | 1.37 | 1.13 | 2.59 | 2.17 | 2.91 |
| **Myocardial infarction** | | | | | | | | | | | | |
| **Longitudinal strain** | | | | | | | | | | | | |
| 1.33 | 2.09 | 1.22 | 1.43 | 1.03 | 1.02 | 1.21 | 1.39 | 1.36 | 1.13 | 2.57 | 2.22 | 1.73 |
| **Circumferential strain** | | | | | | | | | | | | |
| 1.45 | 3.26 | 1.20 | 1.44 | 1.03 | 1.02 | 1.22 | 1.39 | 1.36 | 1.13 | 2.32 | 1.95 | 2.81 |
| **Radial strain** | | | | | | | | | | | | |
| 1.08 | 2.73 | 1.19 | 1.43 | 1.03 | 1.02 | 1.22 | 1.39 | 1.36 | 1.12 | 2.31 | 1.95 | 2.68 |
| **Heart failure** | | | | | | | | | | | | |
| **Longitudinal strain** | | | | | | | | | | | | |
| 1.32 | 2.60 | 1.22 | 1.36 | 1.03 | 1.02 | 1.22 | 1.42 | 1.38 | 1.14 | 2.88 | 2.66 | 2.49 |
| **Circumferential strain** | | | | | | | | | | | | |
| 1.85 | 4.57 | 1.19 | 1.38 | 1.03 | 1.02 | 1.23 | 1.43 | 1.38 | 1.14 | 2.75 | 2.52 | 4.04 |
| **Radial strain** | | | | | | | | | | | | |
| 1.27 | 3.64 | 1.19 | 1.38 | 1.03 | 1.02 | 1.22 | 1.43 | 1.38 | 1.14 | 2.74 | 2.51 | 3.76 |
| **Arrhythmia** | | | | | | | | | | | | |
| **Longitudinal strain** | | | | | | | | | | | | |
| 1.31 | 2.12 | 1.20 | 1.47 | 1.02 | 1.01 | 1.21 | 1.39 | 1.37 | 1.13 | 2.77 | 2.38 | 1.92 |
| **Circumferential strain** | | | | | | | | | | | | |
| 1.57 | 3.55 | 1.18 | 1.49 | 1.02 | 1.01 | 1.21 | 1.40 | 1.37 | 1.13 | 2.62 | 2.19 | 3.06 |
| **Radial strain** | | | | | | | | | | | | |
| 1.12 | 2.92 | 1.18 | 1.48 | 1.02 | 1.01 | 1.21 | 1.39 | 1.37 | 1.13 | 2.62 | 2.19 | 2.91 |
| **Death from any cause** | | | | | | | | | | | | |
| **Longitudinal strain** | | | | | | | | | | | | |
| 1.30 | 2.01 | 1.18 | 1.51 | 1.02 | 1.02 | 1.21 | 1.37 | 1.35 | 1.14 | 2.64 | 2.22 | 1.73 |
| **Circumferential strain** | | | | | | | | | | | | |
| 1.48 | 3.27 | 1.16 | 1.51 | 1.02 | 1.02 | 1.21 | 1.37 | 1.35 | 1.14 | 2.47 | 2.01 | 2.78 |
| **Radial strain** | | | | | | | | | | | | |
| 1.09 | 2.74 | 1.16 | 1.50 | 1.02 | 1.02 | 1.21 | 1.37 | 1.35 | 1.14 | 2.47 | 2.01 | 2.68 |

CoV: coefficient of variation; CV: cardiovascular; CMR: cardiovascular magnetic resonance imaging; BMI: body mass index; HTN: hypertension; LVMi: indexed left ventricular mass; LVEDVi: indexed left ventricular end-diastolic volume; LVEF: left ventricular ejection fraction.

# Supplemental Table 3. Results of Cox proportional hazards regression models to predict a composite CV endpoint by global & CoV strain.

| **Biomarker** | **Model** |  | **HR** | **CI** | **P-value** |  | **△C-index** | **C-index** | **P-value** |  | **NRI(>0) [%]** |  | **△ Brier score** |  | **△AIC** |
| --- | --- | --- | --- | --- | --- | --- | --- | --- | --- | --- | --- | --- | --- | --- | --- |
| CoV_LS_ | 1 |  | 1.21 | 1.15 - 1.28 | <0.001 |  | 0.054 | 0.554 | <0.001 |  | 6.3 (3.7 - 8.4) |  | -0.00003 |  | -48 |
|  | 2 |  | 1.11 | 1.05 - 1.18 | 0.004 |  | 0.002 | 0.716 | <0.001 |  | 2.6 (-0.7 - 5.5) |  | 0.00001 |  | -11 |
|  | 3 |  | 0.95 | 0.90 - 1.01 | 1.00 |  | -0.001 | 0.728 | 0.16 |  | 0.0 (-2.4 - 3.8) |  | 0.00000 |  | 0 |
| GLS | 1 |  | 0.65 | 0.61 - 0.68 | <0.001 |  | 0.090 | 0.590 | <0.001 |  | 17.1 (13.3 - 19.9) |  | -0.00027 |  | -271 |
|  | 2 |  | 0.74 | 0.70 - 0.78 | <0.001 |  | 0.004 | 0.718 | <0.001 |  | 9.1 (6.0 - 12.5) |  | -0.00011 |  | -116 |
|  | 3 |  | 0.77 | 0.71 - 0.82 | <0.001 |  | - | - | - |  | - |  | - |  | - |
| CoV_CS_ | 1 |  | 1.28 | 1.22 - 1.34 | <0.001 |  | 0.043 | 0.543 | <0.001 |  | 9.4 (6.3 - 12.7) |  | -0.00007 |  | -88 |
|  | 2 |  | 1.22 | 1.17 - 1.28 | <0.001 |  | 0.006 | 0.720 | <0.001 |  | 6.4 (3.5 - 9) |  | -0.00003 |  | -62 |
|  | 3 |  | 1.10 | 1.04 - 1.16 | 0.017 |  | 0.001 | 0.729 | <0.001 |  | 2.3 (-0.9 - 5.3) |  | 0.00001 |  | -8 |
| GCS | 1 |  | 0.71 | 0.67 - 0.75 | <0.001 |  | 0.065 | 0.565 | <0.001 |  | 11.8 (8.6 - 15.3) |  | -0.00019 |  | -158 |
|  | 2 |  | 0.77 | 0.73 - 0.81 | <0.001 |  | 0.003 | 0.717 | <0.001 |  | 7.4 (4.4 - 10.2) |  | -0.00011 |  | -89 |
|  | 3 |  | 0.93 | 0.85 - 1.02 | 1.00 |  | - | - | - |  | - |  | - |  | - |
| CoV_RS_ | 1 |  | 1.14 | 1.08 - 1.20 | <0.001 |  | 0.026 | 0.526 | <0.001 |  | 6.6 (3.7 - 9.9) |  | -0.00001 |  | -20 |
|  | 2 |  | 1.12 | 1.06 - 1.18 | <0.001 |  | 0.002 | 0.716 | <0.001 |  | 5.6 (2.2 - 8.2) |  | 0.00000 |  | -16 |
|  | 3 |  | 1.06 | 1.01 - 1.12 | 0.40 |  | 0.001 | 0.728 | 0.80 |  | 2.0 (-1.7 - 4.6) |  | 0.00001 |  | -3 |
| GRS | 1 |  | 0.72 | 0.68 - 0.76 | <0.001 |  | 0.064 | 0.564 | <0.001 |  | 10.8 (7.5 - 14.2) |  | -0.00014 |  | -126 |
|  | 2 |  | 0.78 | 0.74 - 0.83 | <0.001 |  | 0.002 | 0.716 | <0.001 |  | 7.7 (4.4 - 10.4) |  | -0.00009 |  | -70 |
|  | 3 |  | 0.94 | 0.86 - 1.02 | 1.00 |  | - | - | - |  | - |  | - |  | - |

CV: cardiovascular; CoV: coefficient of variation; HR: hazard ratio; CI: confidence interval; NRI(>0): continuous net reclassification improvement; AIC: Akaike information criterion; CoV_LS_: longitudinal strain coefficient of variation; GLS: global longitudinal strain; CoV_CS_: circumferential strain coefficient of variation; GCS: global circumferential strain; CoV_RS_: radial strain coefficient of variation; GRS: global radial strain. *P-value multiplied by Bonferroni correction factor 15.

# Supplemental Table 4. Results of Cox proportional hazards regression models to predict incident MI by global & CoV strain.

| **Biomarker** | **Model** |  | **HR** | **CI** | **P-value** |  | **△C-index** | **C-index** | **P-value** |  | **NRI(>0) [%]** |  | **△ Brier score** |  | **△AIC** |
| --- | --- | --- | --- | --- | --- | --- | --- | --- | --- | --- | --- | --- | --- | --- | --- |
| CoV_LS_ | 1 |  | 1.25 | 1.15 - 1.36 | <0.001 |  | 0.070 | 0.570 | <0.001 |  | 9 (4.2 - 14.3) |  | 0.00000 |  | -23 |
|  | 2 |  | 1.10 | 1.00 - 1.20 | 0.73 |  | 0.002 | 0.724 | <0.001 |  | 3.4 (-1.4 - 8.2) |  | 0.00000 |  | -2 |
|  | 3 |  | 0.99 | 0.90 - 1.10 | 1.00 |  | 0.000 | 0.730 | 0.46 |  | -2 (-3.7 - 7.4) |  | 0.00000 |  | 2 |
| GLS | 1 |  | 0.68 | 0.63 - 0.74 | <0.001 |  | 0.107 | 0.607 | <0.001 |  | 16.2 (11.4 - 21.6) |  | -0.00002 |  | -75 |
|  | 2 |  | 0.82 | 0.75 - 0.89 | <0.001 |  | 0.003 | 0.725 | <0.001 |  | 7.5 (2.9 - 13.2) |  | 0.00000 |  | -18 |
|  | 3 |  | 0.90 | 0.80 - 1.01 | 1.00 |  | - | - | - |  | - |  | - |  | - |
| CoV_CS_ | 1 |  | 1.19 | 1.09 - 1.29 | 0.001 |  | 0.042 | 0.542 | <0.001 |  | 7.8 (2.9 - 12.6) |  | 0.00000 |  | -14 |
|  | 2 |  | 1.12 | 1.03 - 1.21 | 0.081 |  | 0.002 | 0.723 | <0.001 |  | 4.2 (-0.2 - 8.7) |  | 0.00000 |  | -5 |
|  | 3 |  | 1.02 | 0.93 - 1.12 | 1.00 |  | 0.000 | 0.731 | 0.057 |  | -0.4 (-3.8 - 5.7) |  | 0.00000 |  | 2 |
| GCS | 1 |  | 0.74 | 0.68 - 0.81 | <0.001 |  | 0.072 | 0.572 | <0.001 |  | 12.7 (7.6 - 17.3) |  | -0.00001 |  | -43 |
|  | 2 |  | 0.85 | 0.78 - 0.92 | 0.002 |  | 0.001 | 0.723 | 0.012 |  | 5.7 (1.3 - 10.5) |  | -0.00001 |  | -12 |
|  | 3 |  | 0.90 | 0.78 - 1.04 | 1.00 |  | - | - | - |  | - |  | - |  | - |
| CoV_RS_ | 1 |  | 1.06 | 0.97 - 1.16 | 1.00 |  | 0.022 | 0.522 | <0.001 |  | 3.4 (-2.0 - 8.0) |  | 0.00000 |  | 0 |
|  | 2 |  | 1.04 | 0.96 - 1.14 | 1.00 |  | 0.000 | 0.722 | 0.019 |  | 2.1 (-2.9 - 6.8) |  | 0.00000 |  | 1 |
|  | 3 |  | 0.99 | 0.91 - 1.08 | 1.00 |  | 0.000 | 0.730 | 0.24 |  | 1.2 (-3.1 - 6.1) |  | 0.00000 |  | 2 |
| GRS | 1 |  | 0.74 | 0.68 - 0.81 | <0.001 |  | 0.071 | 0.571 | <0.001 |  | 11.7 (7.0 - 16.8) |  | -0.00001 |  | -39 |
|  | 2 |  | 0.84 | 0.77 - 0.93 | 0.005 |  | 0.001 | 0.722 | 0.001 |  | 4.9 (0.0 - 9.4) |  | 0.00000 |  | -11 |
|  | 3 |  | 0.89 | 0.77 - 1.03 | 1.00 |  | - | - | - |  | - |  | - |  | - |

MI: myocardial infarction; CoV: coefficient of variation; HR: hazard ratio; CI: confidence interval; NRI(>0): continuous net reclassification improvement; AIC: Akaike information criterion; CoV_LS_: longitudinal strain coefficient of variation; GLS: global longitudinal strain; CoV_CS_: circumferential strain coefficient of variation; GCS: global circumferential strain; CoV_RS_: radial strain coefficient of variation; GRS: global radial strain. *P-value multiplied by Bonferroni correction factor 15.

# Supplemental Table 5. Results of Cox proportional hazards regression models to predict incident HF by global & CoV strain.

| **Biomarker** | **Model** |  | **HR** | **CI** | **P-value*** |  | **△C-index** | **C-index** | **P-value** |  | **NRI(>0) [%]** |  | **△ Brier score** |  | **△AIC** |
| --- | --- | --- | --- | --- | --- | --- | --- | --- | --- | --- | --- | --- | --- | --- | --- |
| CoV_LS_ | 1 |  | 1.35 | 1.23 - 1.49 | <0.001 |  | 0.067 | 0.567 | <0.001 |  | 12.2 (5.3 - 16.8) |  | -0.00001 |  | -35 |
|  | 2 |  | 1.24 | 1.12 - 1.37 | 0.001 |  | 0.004 | 0.777 | <0.001 |  | 9 (3.5 - 14.9) |  | 0.00000 |  | -15 |
|  | 3 |  | 0.92 | 0.83 - 1.02 | 1.00 |  | 0.001 | 0.824 | 0.24 |  | -2.7 (-12 - 7.2) |  | 0.00000 |  | 0 |
| GLS | 1 |  | 0.48 | 0.44 - 0.52 | <0.001 |  | 0.162 | 0.662 | <0.001 |  | 28.5 (23.2 - 34.5) |  | -0.00008 |  | -269 |
|  | 2 |  | 0.57 | 0.53 - 0.62 | <0.001 |  | 0.019 | 0.792 | <0.001 |  | 20.7 (14.6 - 25.6) |  | -0.00004 |  | -149 |
|  | 3 |  | 0.66 | 0.59 - 0.75 | <0.001 |  | - | - | - |  | - |  | - |  | - |
| CoV_CS_ | 1 |  | 1.73 | 1.62 - 1.83 | <0.001 |  | 0.154 | 0.654 | <0.001 |  | 22.2 (15.2 - 27.5) |  | -0.00001 |  | -204 |
|  | 2 |  | 1.55 | 1.46 - 1.65 | <0.001 |  | 0.031 | 0.804 | <0.001 |  | 14.2 (9.2 - 19.4) |  | 0.00002 |  | -142 |
|  | 3 |  | 1.20 | 1.11 - 1.31 | <0.001 |  | 0.004 | 0.826 | <0.001 |  | 1.5 (-4.6 - 7.7) |  | 0.00002 |  | -15 |
| GCS | 1 |  | 0.47 | 0.44 - 0.51 | <0.001 |  | 0.167 | 0.667 | <0.001 |  | 28.4 (22.5 - 34.3) |  | -0.00011 |  | -267 |
|  | 2 |  | 0.55 | 0.50 - 0.59 | <0.001 |  | 0.029 | 0.802 | <0.001 |  | 21.0 (14.7 - 26.9) |  | -0.00006 |  | -183 |
|  | 3 |  | 0.79 | 0.67 - 0.92 | 0.041 |  | - | - | - |  | - |  | - |  | - |
| CoV_RS_ | 1 |  | 1.56 | 1.45 - 1.69 | <0.001 |  | 0.120 | 0.620 | <0.001 |  | 20.7 (14.6 - 26.3) |  | -0.00001 |  | -105 |
|  | 2 |  | 1.45 | 1.35 - 1.56 | <0.001 |  | 0.020 | 0.793 | <0.001 |  | 15.8 (9.3 - 21.0) |  | 0.00001 |  | -79 |
|  | 3 |  | 1.21 | 1.11 - 1.31 | <0.001 |  | 0.004 | 0.825 | <0.001 |  | 5.2 (-1.3 - 10.8) |  | 0.00002 |  | -17 |
| GRS | 1 |  | 0.45 | 0.40 - 0.50 | <0.001 |  | 0.164 | 0.664 | <0.001 |  | 26.1 (20.5 - 32.5) |  | -0.00009 |  | -227 |
|  | 2 |  | 0.52 | 0.47 - 0.57 | <0.001 |  | 0.024 | 0.797 | <0.001 |  | 21.4 (15.3 - 27.2) |  | -0.00007 |  | -158 |
|  | 3 |  | 0.77 | 0.66 - 0.91 | 0.028 |  | - | - | - |  | - |  | - |  | - |

HF: heart failure; CoV: coefficient of variation; HR: hazard ratio; CI: confidence interval; NRI(>0): continuous net reclassification improvement; AIC: Akaike information criterion; CoV_LS_: longitudinal strain coefficient of variation; GLS: global longitudinal strain; CoV_CS_: circumferential strain coefficient of variation; GCS: global circumferential strain; CoV_RS_: radial strain coefficient of variation; GRS: global radial strain. *P-value multiplied by Bonferroni correction factor 15.

# Supplemental Table 6. Results of Cox proportional hazards regression models to predict incident arrhythmia by global & CoV strain.

| **Biomarker** | **Model** |  | **HR** | **CI** | **P-value** |  | **△C-index** | **C-index** | **P-value** |  | **NRI(>0) [%]** |  | **△ Brier score** |  | **△AIC** |
| --- | --- | --- | --- | --- | --- | --- | --- | --- | --- | --- | --- | --- | --- | --- | --- |
| CoV_LS_ | 1 |  | 1.18 | 1.10 - 1.25 | <0.001 |  | 0.044 | 0.544 | <0.001 |  | 4.7 (1.5 - 8.1) |  | -0.00001 |  | -23 |
|  | 2 |  | 1.09 | 1.02 - 1.16 | 0.23 |  | 0.001 | 0.720 | 0.014 |  | 1.4 (-1.9 - 5.0) |  | 0.00000 |  | -4 |
|  | 3 |  | 0.94 | 0.87 - 1.01 | 1.38 |  | 0.000 | 0.736 | 0.30 |  | 1.6 (-2.2 - 5.3) |  | 0.00000 |  | -1 |
| GLS | 1 |  | 0.65 | 0.61 - 0.69 | <0.001 |  | 0.084 | 0.584 | <0.001 |  | 16.2 (12.5 - 19.1) |  | -0.00013 |  | -181 |
|  | 2 |  | 0.76 | 0.71 - 0.81 | <0.001 |  | 0.002 | 0.721 | <0.001 |  | 7.8 (4.1 - 12.0) |  | -0.00006 |  | -70 |
|  | 3 |  | 0.75 | 0.69 - 0.82 | <0.001 |  | - | - | - |  | - |  | - |  | - |
| CoV_CS_ | 1 |  | 1.29 | 1.22 - 1.37 | <0.001 |  | 0.048 | 0.548 | <0.001 |  | 11 (7.4 - 14.7) |  | -0.00003 |  | -68 |
|  | 2 |  | 1.21 | 1.14 - 1.27 | <0.001 |  | 0.004 | 0.723 | <0.001 |  | 6.3 (2.8 - 9.6) |  | -0.00001 |  | -40 |
|  | 3 |  | 1.11 | 1.03 - 1.18 | 0.044 |  | 0.002 | 0.736 | 0.001 |  | 2.9 (-0.7 - 6.2) |  | 0.00001 |  | -7 |
| GCS | 1 |  | 0.73 | 0.69 - 0.78 | <0.001 |  | 0.059 | 0.559 | <0.001 |  | 9.6 (5.9 - 13.1) |  | -0.00007 |  | -93 |
|  | 2 |  | 0.80 | 0.76 - 0.85 | <0.001 |  | 0.001 | 0.720 | 0.002 |  | 5.5 (2.4 – 9.0) |  | -0.00005 |  | -45 |
|  | 3 |  | 0.98 | 0.88 - 1.09 | 1.00 |  | - | - | - |  | - |  | - |  | - |
| CoV_RS_ | 1 |  | 1.17 | 1.10 - 1.25 | <0.001 |  | 0.036 | 0.536 | <0.001 |  | 7.5 (3.7 - 11.2) |  | -0.00001 |  | -23 |
|  | 2 |  | 1.13 | 1.07 - 1.20 | <0.001 |  | 0.002 | 0.721 | <0.001 |  | 4.5 (1.5 - 7.6) |  | 0.00000 |  | -15 |
|  | 3 |  | 1.07 | 1.01 - 1.14 | 0.30 |  | 0.001 | 0.735 | 0.004 |  | 2.6 (-1.0 - 6.2) |  | 0.00001 |  | -3 |
| GRS | 1 |  | 0.75 | 0.70 - 0.80 | <0.001 |  | 0.058 | 0.558 | <0.001 |  | 8.8 (5 - 12.4) |  | -0.00005 |  | -72 |
|  | 2 |  | 0.81 | 0.76 - 0.87 | <0.001 |  | 0.001 | 0.719 | 0.38 |  | 6.0 (2.1 - 9.5) |  | -0.00004 |  | -34 |
|  | 3 |  | 0.98 | 0.89 - 1.09 | 1.00 |  | - | - | - |  | - |  | - |  | - |

CoV: coefficient of variation; HR: hazard ratio; CI: confidence interval; NRI(>0): continuous net reclassification improvement; AIC: Akaike information criterion; CoV_LS_: longitudinal strain coefficient of variation; GLS: global longitudinal strain; CoV_CS_: circumferential strain coefficient of variation; GCS: global circumferential strain; CoV_RS_: radial strain coefficient of variation; GRS: global radial strain. *P-value multiplied by Bonferroni correction factor 15.

# Supplemental Table 7. Results of Cox proportional hazards regression models to predict death from any cause by global & CoV strain.

| **Biomarker** | **Model** |  | **HR** | **CI** | **P-value*** |  | **△C-index** | **C-index** | **P-value** |  | **NRI(>0) [%]** |  | **△ Brier score** |  | **△AIC** |
| --- | --- | --- | --- | --- | --- | --- | --- | --- | --- | --- | --- | --- | --- | --- | --- |
| CoV_LS_ | 1 |  | 1.04 | 0.97 - 1.12 | 1.00 |  | 0.004 | 0.504 | <0.001 |  | 1 (-3.1 - 4.6) |  | 0.00000 |  | 1 |
|  | 2 |  | 0.97 | 0.90 - 1.05 | 1.00 |  | 0.000 | 0.723 | 0.24 |  | -1.6 (-4.5 - 6.3) |  | 0.00000 |  | 2 |
|  | 3 |  | 0.89 | 0.82 - 0.97 | 0.12 |  | 0.001 | 0.728 | 0.50 |  | 0.4 (-3.6 - 4.4) |  | 0.00000 |  | -5 |
| GLS | 1 |  | 0.72 | 0.67 - 0.77 | <0.001 |  | 0.094 | 0.594 | <0.001 |  | 13.6 (9.4 - 17.5) |  | -0.00002 |  | -84 |
|  | 2 |  | 0.85 | 0.79 - 0.91 | <0.001 |  | 0.004 | 0.727 | <0.001 |  | 6.6 (1.7 - 11.1) |  | 0.00000 |  | -17 |
|  | 3 |  | 0.84 | 0.77 - 0.93 | 0.007 |  | - | - | - |  | - |  | - |  | - |
| CoV_CS_ | 1 |  | 1.22 | 1.14 - 1.30 | <0.001 |  | 0.031 | 0.531 | <0.001 |  | 8 (3.5 - 12) |  | -0.00001 |  | -29 |
|  | 2 |  | 1.13 | 1.06 - 1.20 | 0.002 |  | 0.000 | 0.723 | 0.10 |  | 3.3 (-0.3 - 7) |  | 0.00000 |  | -12 |
|  | 3 |  | 1.10 | 1.02 - 1.19 | 0.18 |  | -0.001 | 0.724 | 0.31 |  | 2.8 (-1.7 - 7.1) |  | 0.00000 |  | -4 |
| GCS | 1 |  | 0.82 | 0.76 - 0.88 | <0.001 |  | 0.052 | 0.552 | <0.001 |  | 6.5 (2.2 - 10.8) |  | -0.00001 |  | -28 |
|  | 2 |  | 0.90 | 0.84 - 0.97 | 0.075 |  | 0.001 | 0.725 | 0.003 |  | 3.9 (-0.5 - 8.7) |  | -0.00001 |  | -6 |
|  | 3 |  | 1.04 | 0.92 - 1.17 | 1.00 |  | - | - | - |  | - |  | - |  | - |
| CoV_RS_ | 1 |  | 1.16 | 1.08 - 1.24 | <0.001 |  | 0.021 | 0.521 | <0.001 |  | 6.2 (1.7 - 10.6) |  | 0.00000 |  | -15 |
|  | 2 |  | 1.11 | 1.04 - 1.19 | 0.029 |  | 0.000 | 0.723 | 0.12 |  | 2.9 (-0.6 - 6.6) |  | 0.00000 |  | -7 |
|  | 3 |  | 1.08 | 1.01 - 1.16 | 0.31 |  | 0.000 | 0.725 | 0.065 |  | 1.5 (-2.4 - 5.8) |  | 0.00000 |  | -3 |
| GRS | 1 |  | 0.83 | 0.77 - 0.89 | <0.001 |  | 0.050 | 0.550 | <0.001 |  | 7.2 (3.1 - 11.2) |  | -0.00001 |  | -23 |
|  | 2 |  | 0.91 | 0.84 - 0.98 | 0.15 |  | 0.001 | 0.724 | <0.001 |  | 3.5 (-1.1 - 8) |  | -0.00001 |  | -5 |
|  | 3 |  | 1.01 | 0.90 - 1.13 | 1.00 |  | - | - | - |  | - |  | - |  | - |

CoV: coefficient of variation; HR: hazard ratio; CI: confidence interval; NRI(>0): continuous net reclassification improvement; AIC: Akaike information criterion; CoV_LS_: longitudinal strain coefficient of variation; GLS: global longitudinal strain; CoV_CS_: circumferential strain coefficient of variation; GCS: global circumferential strain; CoV_RS_: radial strain coefficient of variation; GRS: global radial strain. *P-value multiplied by Bonferroni correction factor 15.

**Figures**

\


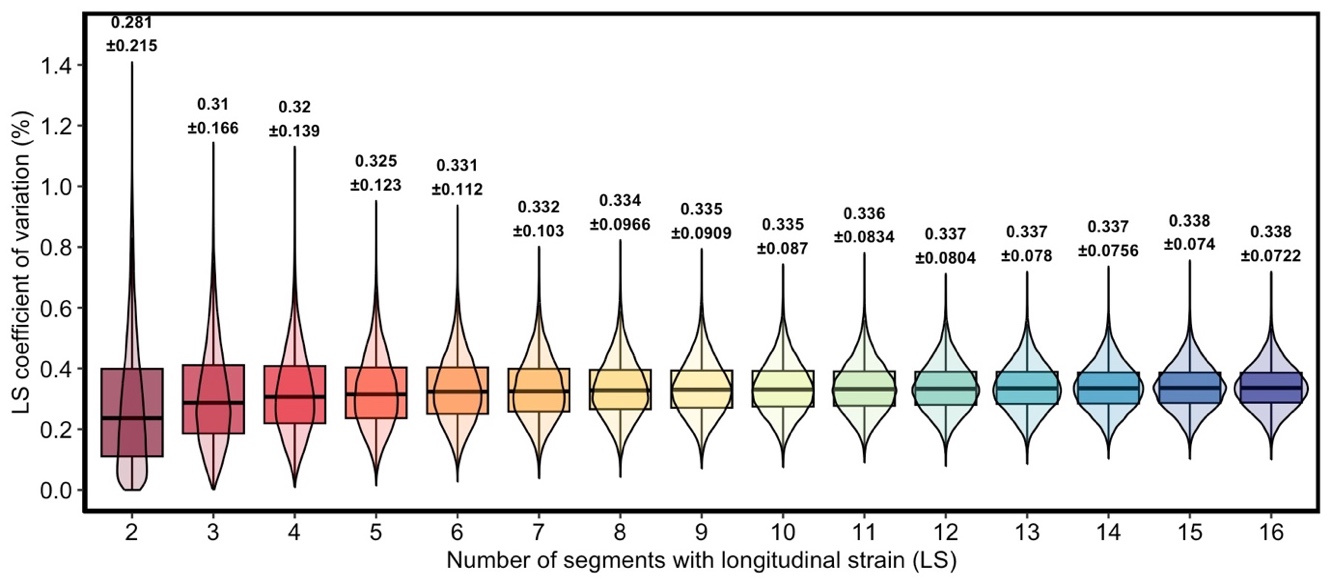

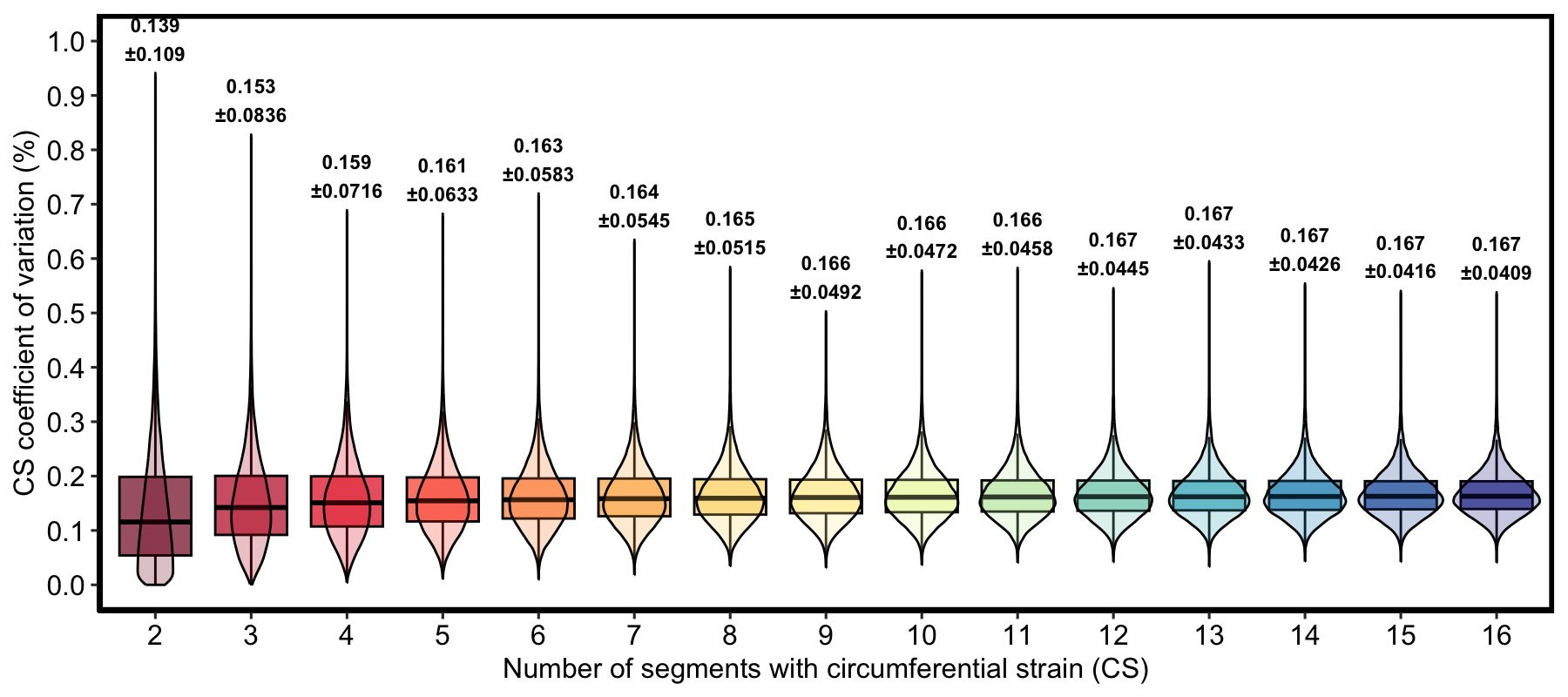


**A)**

**Longitudinal strain (LS)**

**Circumferential strain (CS)**


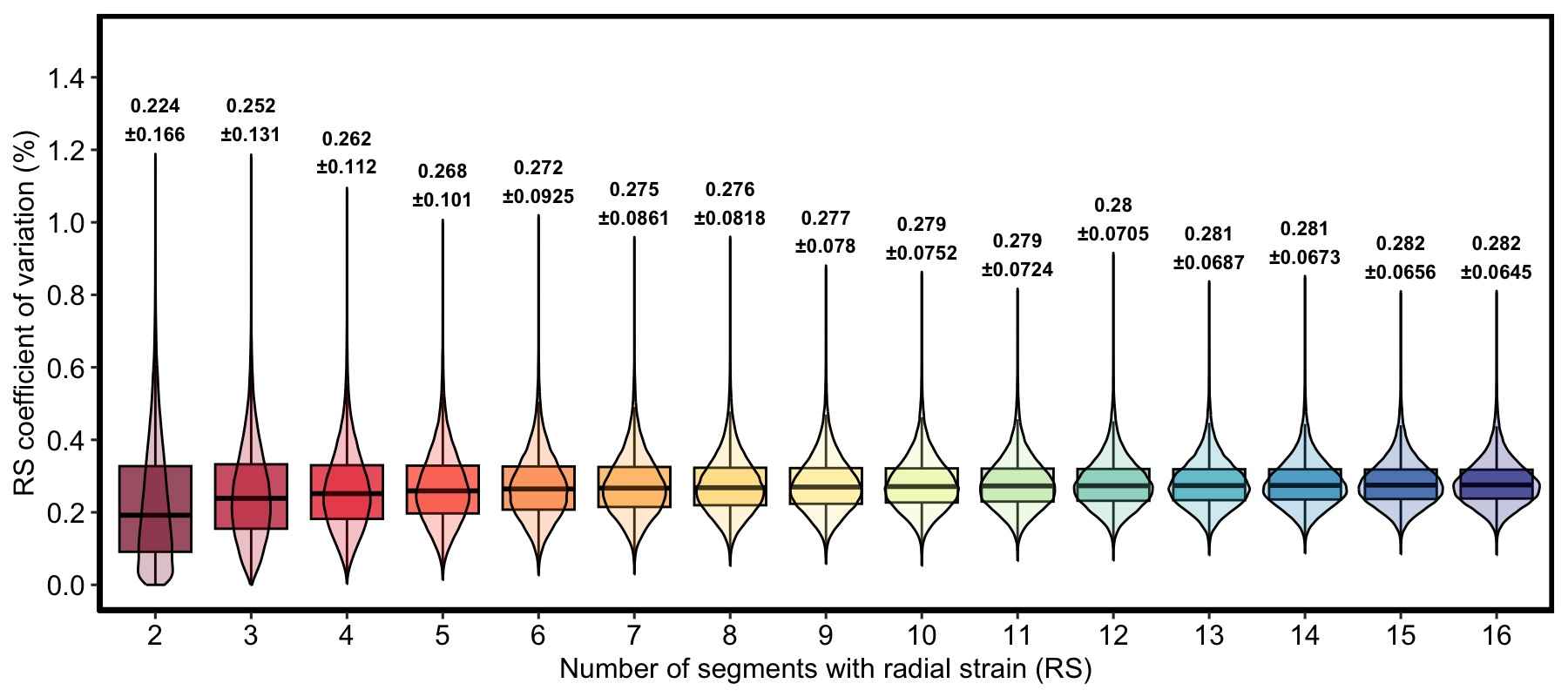

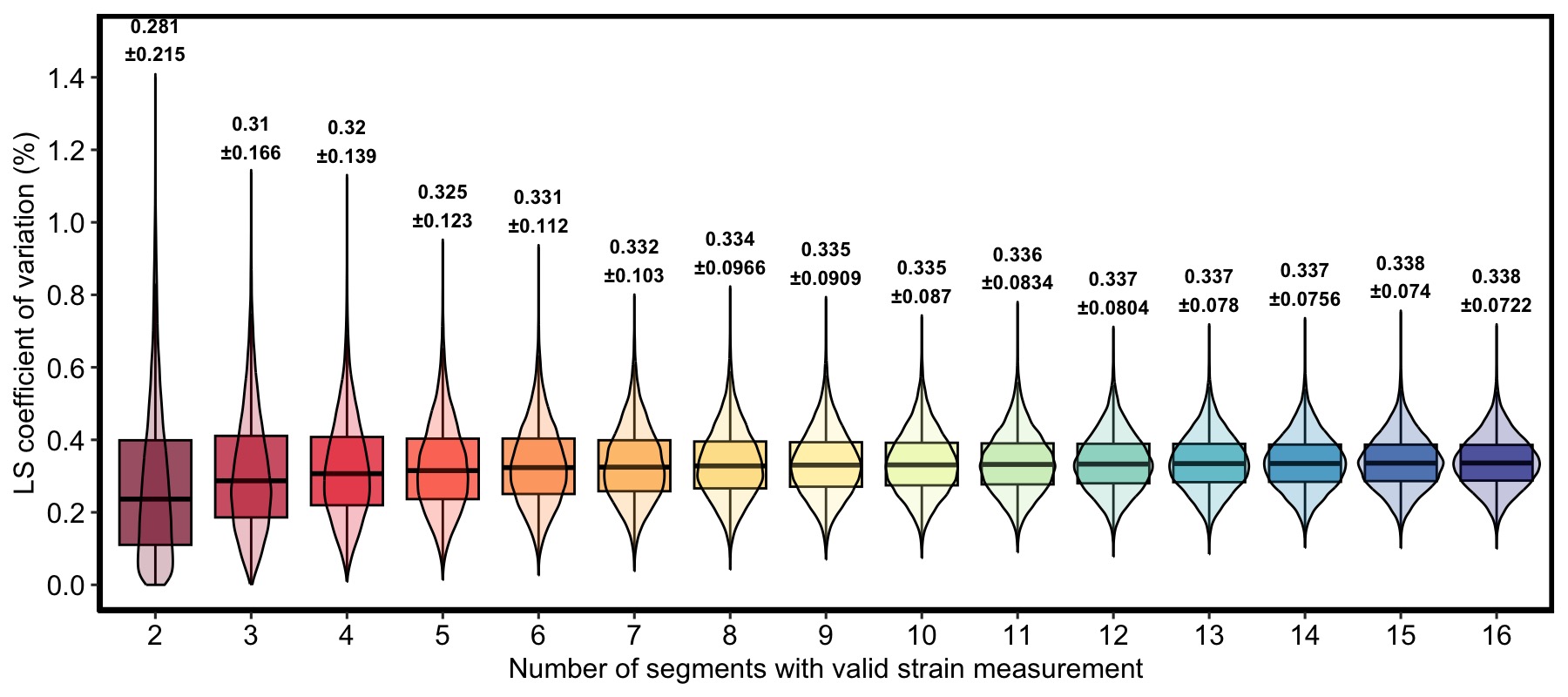


**Radial strain (RS)**

**C)**

**B)**

# Supplemental Figure 1. Strain CoV distribution, stratified by number of valid LV segments.

The central tendency and spread of CoV started to deviate and increase with ≤6 segments for *CoV_LS_, CoV_CS_* and *CoV_RS_.*

Data presented as mean ± standard deviation (SD) and a combined violin-box plot. LV: left ventricle; CoV_LS_: longitudinal strain coefficient of variation; CoV_CS_: circumferential strain coefficient of variation; CoV_RS_: radial strain coefficient of variation.


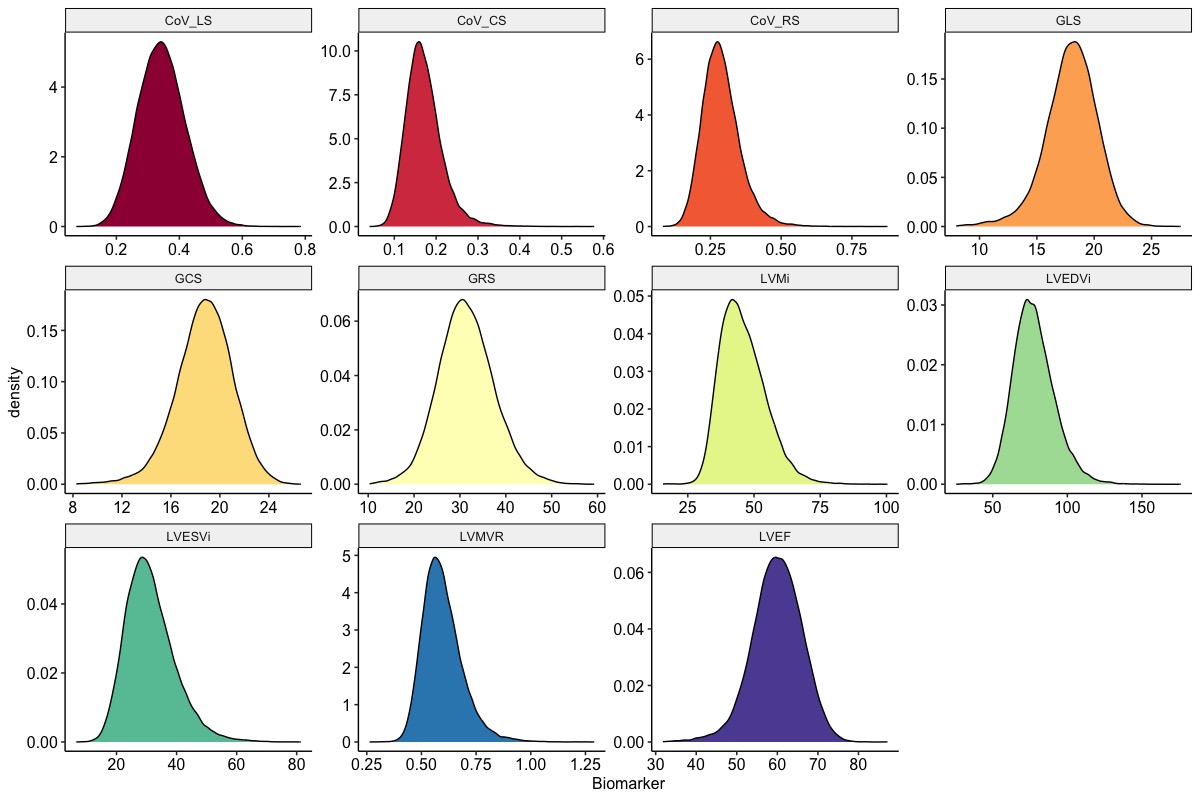


**A)**


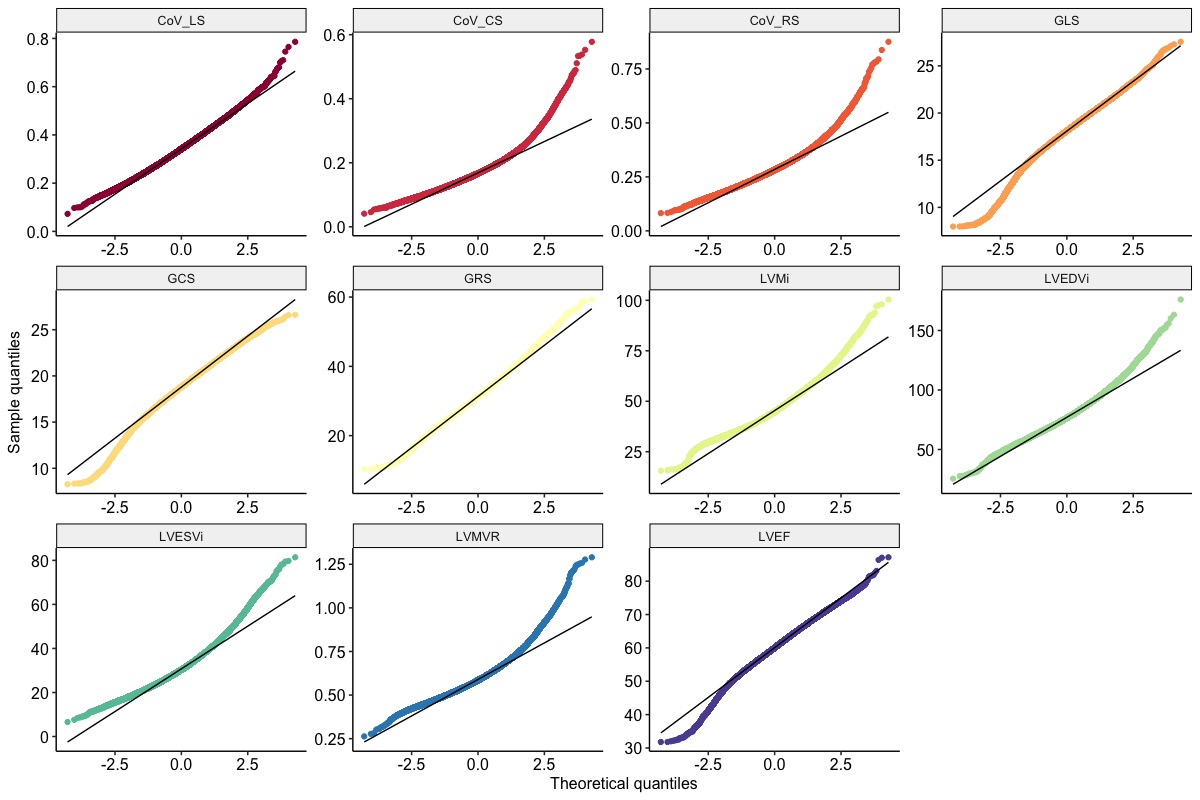


**B)**

# Supplemental Figure 2. CMR biomarker A) density and B) QQ plots.

Overall, *CoV_LS_, CoV_CS_* and *CoV_RS_* had a right-skewed distribution whereas *GLS, GCS* and *GRS* were normally distributed.

CoV_LS_: longitudinal strain coefficient of variation; CoV_CS_: circumferential strain coefficient of variation; CoV_RS_: radial strain coefficient of variation; GLS: global longitudinal strain; GCS: global circumferential strain; GRS: global radial strain; LVMi: indexed left ventricular mass; LVEDVi: indexed left ventricular end-diastolic volume; LVESVi: indexed left ventricular end-systolic volume; LVMVR: left ventricular mass to volume ratio; LVEF: left ventricular ejection fraction; CMR: cardiovascular magnetic resonance imaging.

# Supplemental Figure 3. Incident composite CV endpoint residual *vs.* time plots.

In the fully adjusted Cox regression model for the composite CV endpoint, assumption of proportional hazards is supported for *CoV_LS_, CoV_CS_, CoV_RS,_ GLS, GCS, GRS* and their covariates graphically.

LVMi: indexed left ventricular mass; LVEDVi: indexed left ventricular end-diastolic volume; LVEF: left ventricular ejection fraction; CoV_LS_: longitudinal strain coefficient of variation; CoV_CS_: circumferential strain coefficient of variation; CoV_RS_: radial strain coefficient of variation; GLS: global longitudinal strain; GCS: global circumferential strain; GRS: global radial strain.

# Supplemental Figure 4. Incident myocardial infarction residual *vs.* time plots.

In the fully adjusted Cox regression model for incident myocardial infarction, assumption of proportional hazards is supported for *CoV_LS_, CoV_CS_, CoV_RS,_ GLS, GCS, GRS* and their covariates graphically.

LVMi: indexed left ventricular mass; LVEDVi: indexed left ventricular end-diastolic volume; LVEF: left ventricular ejection fraction; CoV_LS_: longitudinal strain coefficient of variation; CoV_CS_: circumferential strain coefficient of variation; CoV_RS_: radial strain coefficient of variation; GLS: global longitudinal strain; GCS: global circumferential strain; GRS: global radial strain.

# Supplemental Figure 5. Incident heart failure residual *vs.* time plots.

In the fully adjusted Cox regression model for incident heart failure, assumption of proportional hazards is supported for *CoV_LS_, CoV_CS_, CoV_RS,_ GLS, GCS, GRS* and their covariates graphically.

LVMi: indexed left ventricular mass; LVEDVi: indexed left ventricular end-diastolic volume; LVEF: left ventricular ejection fraction; CoV_LS_: longitudinal strain coefficient of variation; CoV_CS_: circumferential strain coefficient of variation; CoV_RS_: radial strain coefficient of variation; GLS: global longitudinal strain; GCS: global circumferential strain; GRS: global radial strain.

# Supplemental Figure 6. Incident arrhythmia residual *vs.* time plots.

In the fully adjusted Cox regression model for incident arrhythmia, assumption of proportional hazards is supported for *CoV_LS_, CoV_CS_, CoV_RS,_ GLS, GCS, GRS* and their covariates graphically.

LVMi: indexed left ventricular mass; LVEDVi: indexed left ventricular end-diastolic volume; LVEF: left ventricular ejection fraction; CoV_LS_: longitudinal strain coefficient of variation; CoV_CS_: circumferential strain coefficient of variation; CoV_RS_: radial strain coefficient of variation; GLS: global longitudinal strain; GCS: global circumferential strain; GRS: global radial strain.

# Supplemental Figure 7. Death from any cause residual *vs.* time plots.

In the fully adjusted Cox regression model for death from any cause, assumption of proportional hazards is supported for *CoV_LS_, CoV_CS_, CoV_RS,_ GLS, GCS, GRS* and their covariates graphically.

LVMi: indexed left ventricular mass; LVEDVi: indexed left ventricular end-diastolic volume; LVEF: left ventricular ejection fraction; CoV_LS_: longitudinal strain coefficient of variation; CoV_CS_: circumferential strain coefficient of variation; CoV_RS_: radial strain coefficient of variation; GLS: global longitudinal strain; GCS: global circumferential strain; GRS: global radial strain.

# Supplemental Figure 8. Study flowchart, summarizing UKBB participant inclusion criteria.

#
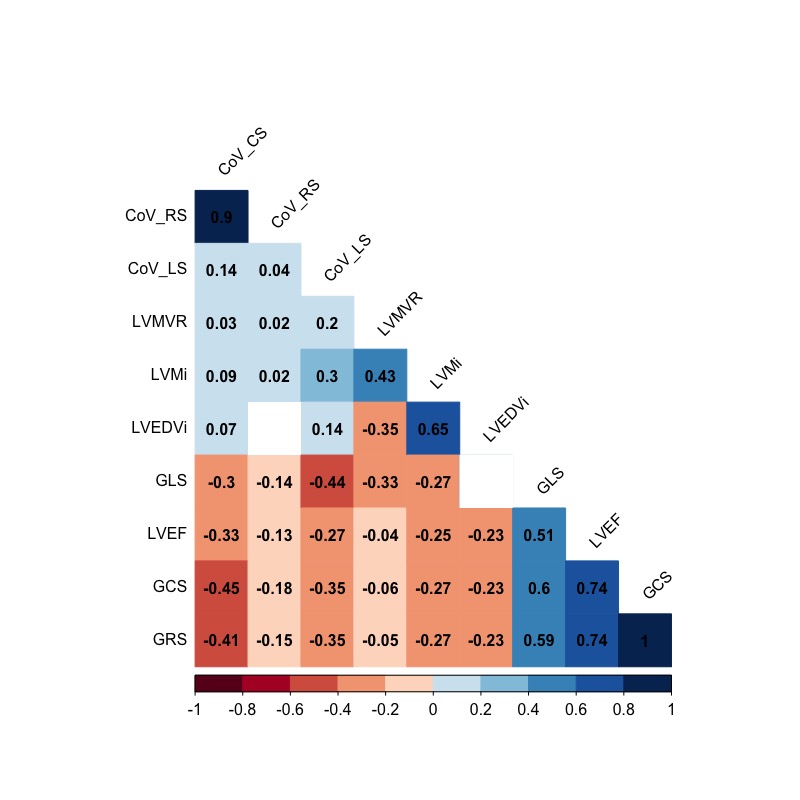
Supplemental Figure 9. Correlogram of CMR biomarkers, including strain CoV.

Strain CoV had at most moderate negative correlation with global strain and LVEF. Global strain was more strongly correlated with LV mass and volume in comparison to strain CoV.

CoV_CS_: circumferential strain coefficient of variation; CoV_RS_: radial strain coefficient of variation; CoV_LS_: longitudinal strain coefficient of variation; LVMVR: left ventricular mass to volume ratio; LVMi: indexed left ventricular mass; LVEDVi: indexed left ventricular end-diastolic volume; GLS: global longitudinal strain; LVEF: left ventricular ejection fraction; GCS: global circumferential strain; GRS: global radial strain; CMR: cardiovascular magnetic resonance imaging.
